# A glimpse into *Oomycota* diversity in freshwater lakes and adjacent forests using a metabarcoding approach

**DOI:** 10.1038/s41598-025-01727-3

**Published:** 2025-05-31

**Authors:** Hossein Masigol, Marcel Dominik Solbach, Mohammad Javad Pourmoghaddam, Reza Ahadi, Reza Mostowfizadeh-Ghalamfarsa, Seyedeh Roksana Taheri, Sven Patrik Tobias-Hünefeldt, Michael Bonkowski, Hans-Peter Grossart

**Affiliations:** 1 Plankton and Microbial Ecology, Leibniz Institute for Freshwater Ecology and Inland Fisheries (IGB), Neuglobsow, Germany; 2 Department of Biology, Terrestrial Ecology, Institute of Zoology, University of Cologne, Cologne, Germany; 3 Department of Plant Protection, Faculty of Agricultural Sciences, University of Guilan, Rasht, Iran; 4 Department of Plant Protection, Faculty of Agriculture, Azarbaijan Shahid Madani University, Tabriz, Iran; 5 Department of Plant Protection, School of Agriculture, Shiraz University, Shiraz, Iran; 6 Dept. of Microbiology and Biotechnology, University of Hamburg, Ohnhorststraße 18, 22609 Hamburg, Germany; 7 Institute of Biochemistry and Biology, Potsdam University, Maulbeerallee 2, D-14469 Potsdam, Germany

**Keywords:** Freshwater *Oomycota*, Metabarcoding, *Peronosporales*, *Pythium*, *Saprolegniales*, Limnology, Biodiversity

## Abstract

*Oomycota*, a diverse group of fungus-like protists, play key ecological roles in aquatic and terrestrial ecosystems, yet their habitat-specific diversity and distribution remain poorly understood. This study investigates the diversity of two major *Oomycota* classes, *Saprolegniomycetes* and *Peronosporomycetes*, in two freshwater lakes and their adjacent forests in northeastern Germany. Using a combination of targeted metabarcoding and traditional isolation techniques, we analyzed samples from six habitats, including soil (forest), rotten leaves (forest and shoreline), sediments (shoreline), and surface waters (littoral and pelagic zones). Metabarcoding revealed 401 *Oomycota* OTUs, with *Pythium*, *Globisporangium*, and *Saprolegnia* as dominant genera. Culture-based methods identified 110 strains, predominantly from surface water and sediment, with *Pythium sensu lato* and *Saprolegnia* as the most frequent taxa. Alpha and beta diversity analyses highlighted distinct community structures influenced by lake and habitat type, with significant co-occurrence of *Saprolegniomycetes* and *Peronosporomycetes* across habitats. This study provides the first comprehensive metabarcoding-based exploration of *Oomycota* biodiversity in interconnected freshwater and terrestrial ecotones, uncovering previously unrecognized patterns of habitat-specific diversity.

**Supplementary Information:**

The online version contains supplementary material available at 10.1038/s41598-025-01727-3.

## Introduction

*Oomycota*, a diverse group of fungus-like protists, play essential roles in both aquatic and terrestrial ecosystems, ranging from saprotrophic decomposers to plant and animal pathogens. Despite their global ecological and economic importance, our understanding of their distribution and diversity across habitats remains limited, largely due to a historical focus on specific taxa in particular environments^1–3^. While it is well-established that the classes *Saprolegniomycetes* and *Peronosporomycetes* inhabit both freshwater and terrestrial environments, research has predominantly focused on the first in freshwater systems and the latter in soils. *Saprolegniomycetes* have been extensively studied as pathogens of amphibians and fish (e.g., *Saprolegnia parasitica*), and crayfish (e.g., *Aphanomyces astaci*)^4–6^, but there are, albeit fewer, several studies examining their occurrence in soil environments^7,8^. A well-documented case of *Saprolegniomycetes* in terrestrial environments is *Aphanomyces euteiches*, which thrives in moist soils and primarily causes root rot disease in leguminous plants^9^. Conversely, while members of *Peronosporomycetes* are infamous for their plant pathogenic roles in soil environments - such as *Pythium ultimum*^10^ (now *Globisporangium ultimum*) and *Phytophthora infestans*^11^, both with broad host ranges - some studies have explored their presence in freshwater ecosystems^12–14^, though such instances remain comparatively less thoroughly investigated. This uneven focus across freshwater and terrestrial ecosystems has resulted in a limited understanding of the true diversity, co-occurrence, and ecological roles of *Saprolegniomycetes* and *Peronosporomycetes* across these habitats.

In addition to the uneven research focus across freshwater and terrestrial ecosystems, several other factors contribute to our limited understanding of *Saprolegniomycetes* and *Peronosporomycetes* diversity and ecological roles. First, research has long relied on culture-based techniques^15^, which tend to favor fast-growing and abundant taxa, and miss rare or unculturable ones. Second, these methods are highly selective, typically isolating only certain genera while overlooking others^16,17^, which complicate comparative studies and limits insights into the distribution of *Saprolegniomycetes* and *Peronosporomycetes* across different environments. However, recently developed metabarcoding approaches have begun to overcome these challenges. Unlike culture-based methods, metabarcoding enables the detection of both fast-growing and rare/unculturable taxa, providing a more comprehensive view of microbial diversity. The application of metabarcoding to study *Saprolegniomycetes* and *Peronosporomycetes* is relatively recent, with most studies thus far focusing on soil environments^18,19^ and only a limited number addressing freshwater ecosystems^20^. This emerging technique holds promise for broadening our understanding on environmental ranges and functions of these groups across terrestrial and aquatic habitats.

Therefore, in this study, we aimed to investigate the diversity of *Saprolegniomycetes* and *Peronosporomycetes* in two distinct freshwater lakes (NE Germany), namely Grosse Fuchskuhle and Grosser Stechlinsee, and their adjacent forests. Forested areas (primarily beech and pine trees) encircle both lakes, and their watersheds remain largely unaffected by human activities. Lake Grosse Fuchskuhle is a naturally acidic bog lake while Grosser Stechlinsee is a dimictic calcareous lake. We used (I) a targeted metabarcoding approach and (II) classic isolation techniques. To our knowledge, this is the first study exploring *Oomycota* biodiversity in freshwater lakes using a targeted metabarcoding approach, complemented by samples from surrounding forest areas. Our findings reveal the co-occurrence of both *Saprolegniomycetes* and *Peronosporomycetes* across freshwater and forest environments, providing new insights into the diversity and distribution of *Oomycota* and their ecological roles in interconnected ecosystems.

## Results

### Overall diversity from the metabarcoding approach

**Fig. 1:**
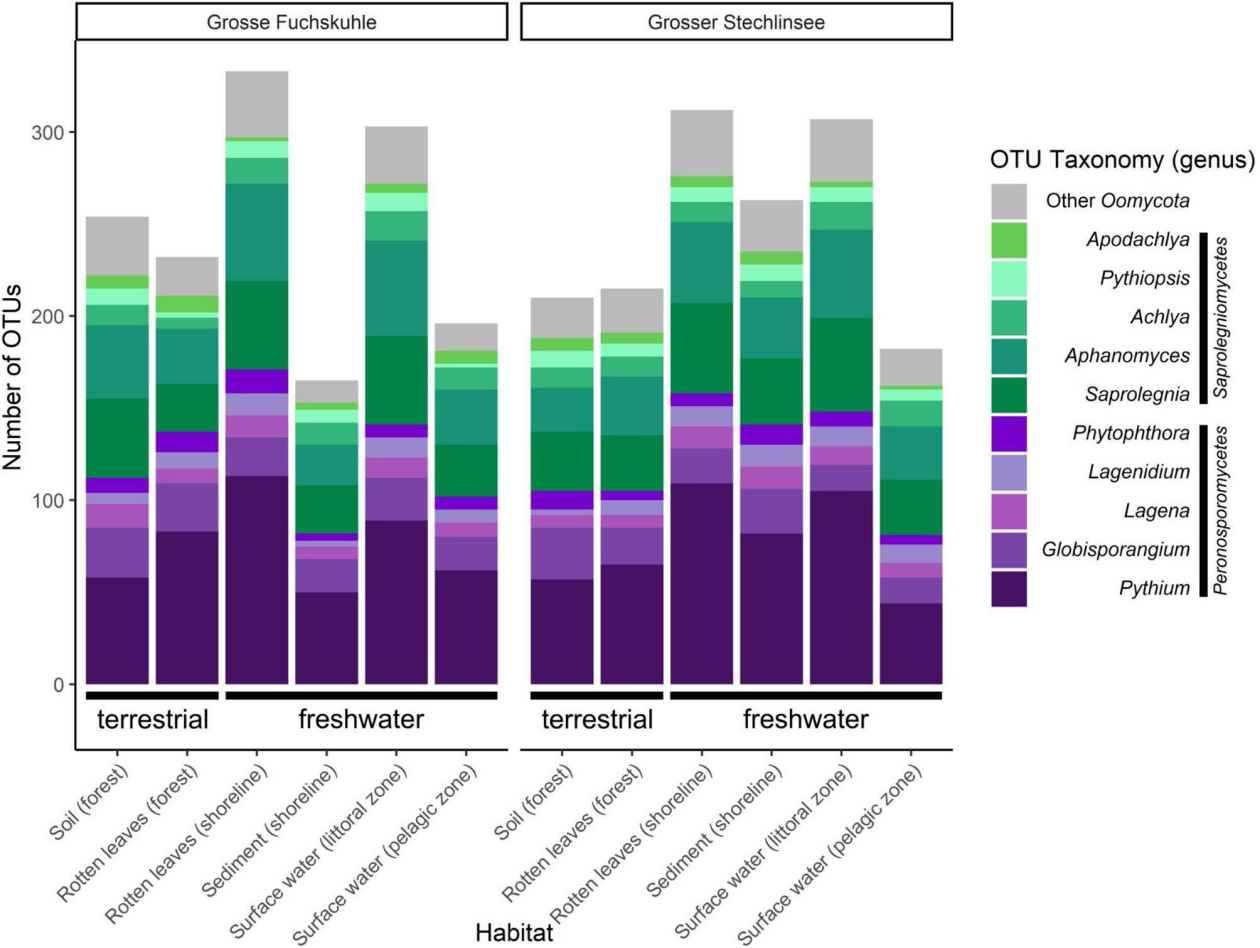
The abundance of detected OTUs belonging to the 10 most common genera from six habitats from Grosse Fuchskuhle and Grosser Stechlinsee lakes (NE Germany) using the metabarcoding approach. Remaining genera are summarized in “Other *Oomycota*”.

**Fig. 2:**
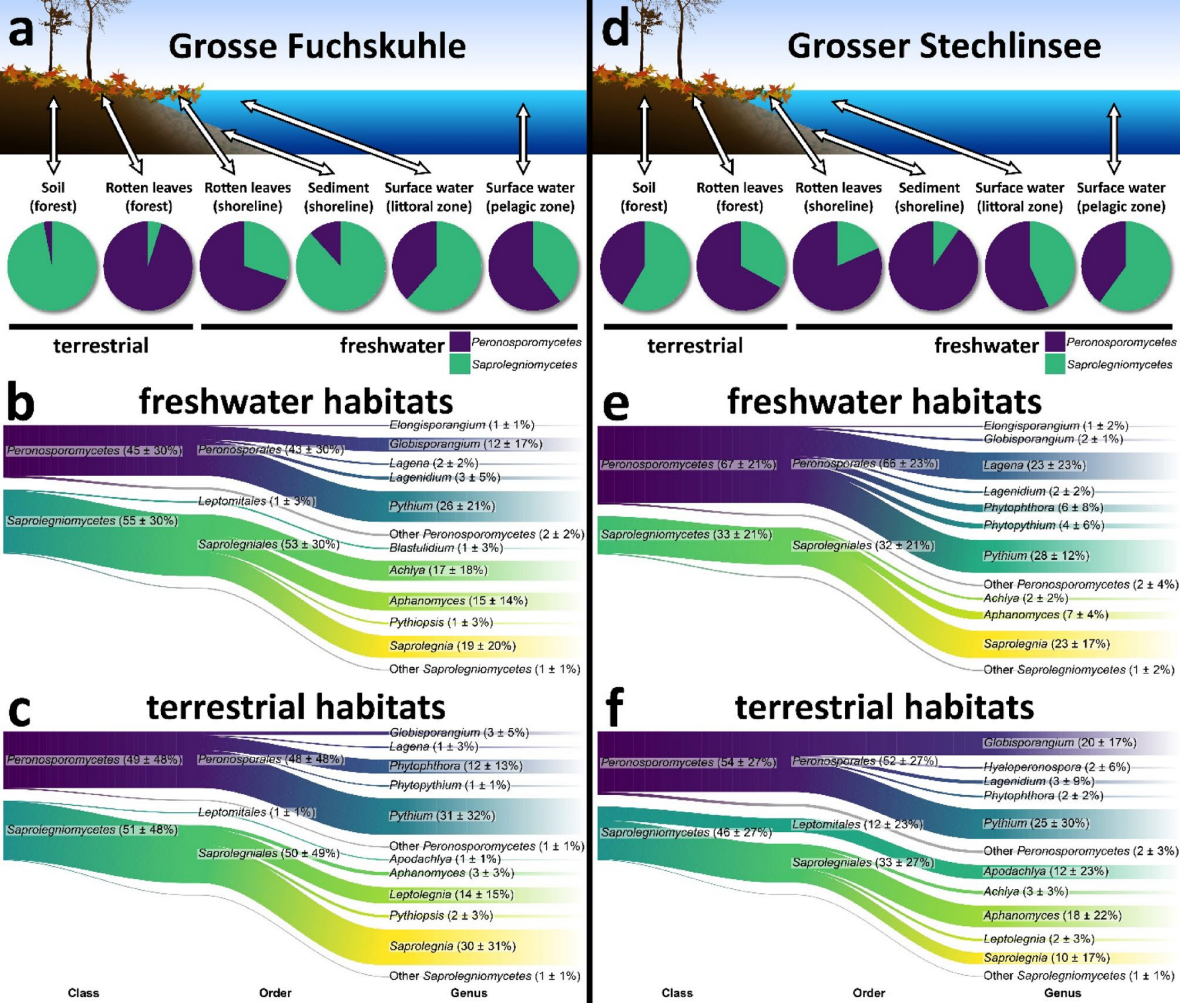
The relative abundance of detected *Oomycota* OTUs across the aquatic-terrestrial ecotones from Lakes Grosse Fuchskuhle (left) and Grosser Stechlinsee (right) using a metabarcoding approach. (a and d) Relative contribution of *Saprolegniomycetes* (*Saprolegniales* and *Leptomitales*) and *Peronosporomycetes* (*Peronosporales* and *Paralagenidiales*) to the diversity of six habitats in each lake. (b and e) Sankey diagrams showing the relative contribution of sequences to the overall taxonomic diversity of freshwater habitats in lakes Grosse Fuchskuhle and Grosser Stechlinsee. (c and f) Sankey diagrams showing the relative sequence contribution to the overall taxonomic diversity of terrestrial habitats from Grosse Fuchskuhle and Grosser Stechlinsee. Taxonomic assignment is based on the best hit by BLAST. Numbers represent sequence read percentages. The digital drawings in (a) and (d) were drawn by Adobe Photoshop 21 (https://www.adobe.com/products/photoshop.html).

The OTU database contained 401 *Oomycota* OTUs after assembly and quality filtering, representing ~ 1.2 million Illumina reads. Samples from the rotten leaves (shoreline) and surface water (littoral) from both lakes showed the highest number of OTUs while the lowest OTU numbers were detected from sediment (shoreline) (Grosse Fuchskuhle) and surface water (pelagic) (Grosser Stechlinsee) (Fig. 1). *Pythium sensu lato* (including *Globisporangium*, and *Pythium sensu stricto*) was the most abundant and diverse detected taxon across all habitats, followed by *Saprolegnia*, *Aphanomyces*, and *Achlya* (Fig. 1, Supplementary Material 1). Analyzing the six habitats of both lakes individually, *Saprolegniomycetes* and *Peronosporomycetes* always occurred together, although in different abundances (Figs. 1 and 2a and d, Supplementary Figure S1). For instance, in Grosse Fuchskuhle, rotten leaves (forest and shoreline) and surface water (pelagic) were more dominated by *Peronosporomycetes* (Fig. 2a). Similarly, rotten leaves (forest and shoreline), sediments (shoreline), and surface water (littoral) habitats in Grosser Stechlinsee contained more *Peronosporomycetes* than *Saprolegniomycetes* (Fig. 2d). At both lakes sites, the soil (forest) was dominated by *Saprolegniomycetes*.

Similar composition patterns of genera emerged when dividing habitats into freshwater and terrestrial. *Pythium sensu lato* and *Saprolegnia* were the most commonly detected OTUs from freshwater habitats of both lakes (Fig. 2b and e). Additionally, we unexpectedly observed ~ 23% of detected OTUs associated with *Lagena* (*Peronosporomycetes*) in freshwater habitats of Grosser Stechlinsee. Terrestrial habitats of both lakes, similar to freshwater ones, were dominated by *Pythium sensu lato* as the most common OTUs (Fig. 2c and f). High occurrence of OTUs associated with *Saprolegnia* (~ 30%) and *Leptolegnia* (~ 14%) was observed in the terrestrial habitats from Grosse Fuchskuhle. Moreover, high occurrence of *Apodachlya*, the only genus which does not belong to *Saprolegniales* or *Peronosporales*, was only observed in the terrestrial habitats of Grosser Stechlinsee.

### Alpha and beta diversity

**Fig. 3:**
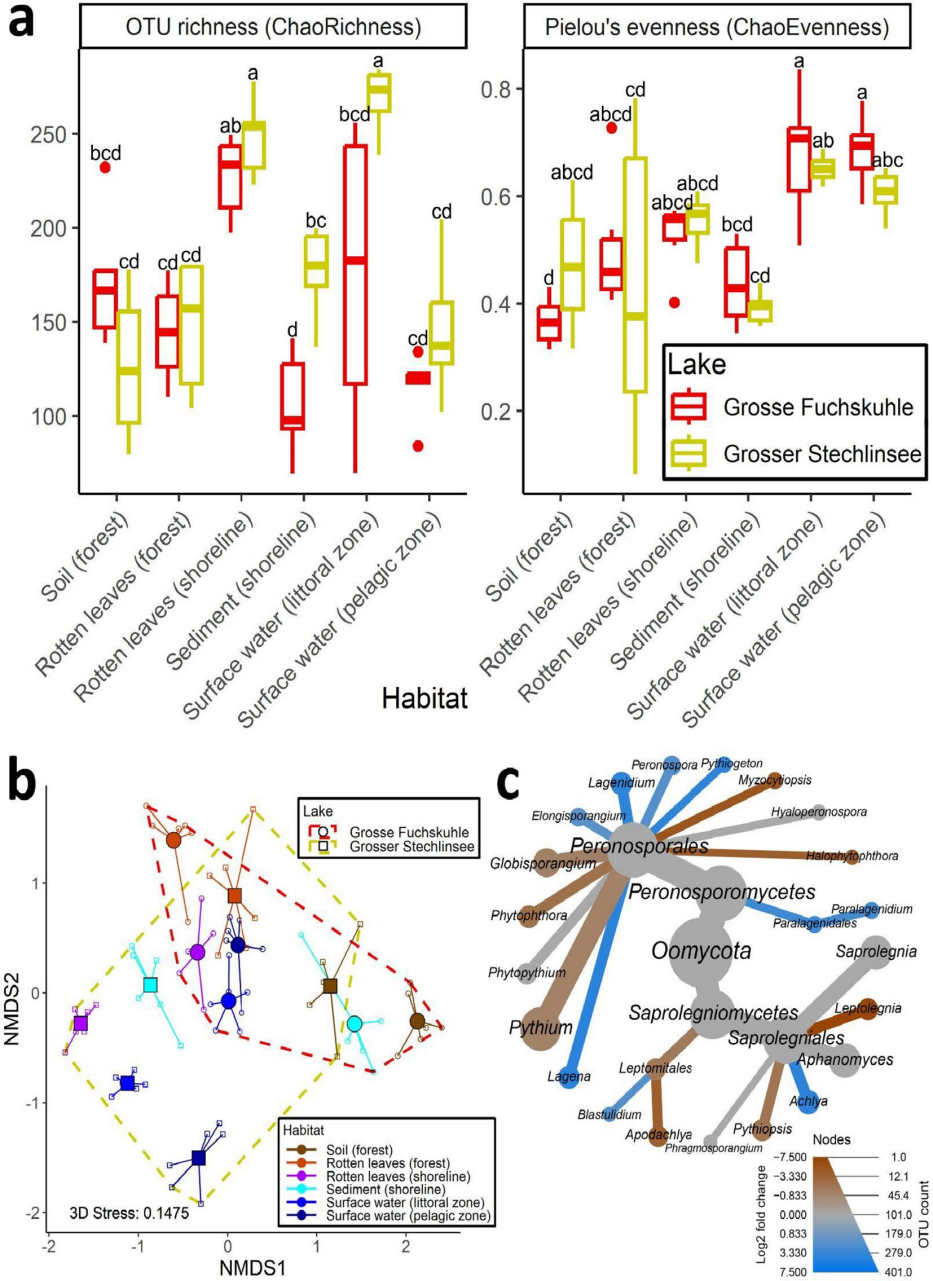
Alpha and beta diversity analysis based on six habitats from Grosse Fuchskuhle and Grosser Stechlinsee and DESeq2 analysis. (a) Boxplots displaying alpha diversity metrics (OTU richness and Pielou’s evenness index). Letters above the boxplots are derived from pairwise comparisons using Tukey’s honest significance test (0.05 significance level). (b) NMDS plot based on Bray-Curtis dissimilarities. Replicates (small symbols) from the same sampling sites were grouped, their centroids (large symbols) were displayed, and sampling sites were differentiated by habitat (color) and lake (symbol and hull). (c) Metacoder heat tree displaying differentially abundant genera between freshwater and terrestrial habitats from DESeq2 analysis. Genera in blue are more abundant in freshwater habitats/less abundant in terrestrial habitats, and genera in brown are more abundant in terrestrial habitats/less abundant in freshwater habitats. Thickness of the branches refers to the observed number of OTUs of the respective taxon.

Alpha diversity differed greatly between the sampling sites (Fig. 3a). Specifically, ChaoRichness differed between both lakes (ANOVA: F_1, 60_ = 11.9410, *p* = 0.001) and habitats (F_5, 60_ = 20.0158, *p* < 0.001). Moreover, *Oomycota* richness did not behave equally for both lakes and the habitats (lake: habitat interaction: F_5, 60_ = 5.7096, *p* < 0.001), suggesting that both lake properties and specific habitat types contribute to variations in *Oomycota* richness. The remaining alpha diversity metrics (exponential Shannon index/ChaoShannon, inverse Simpson index/ChaoSimpson, Supplementary Figure S2) and Pielou’s evenness index/ChaoEvenness (Fig. 3a) differed between habitats (ChaoShannon: F_5, 60_ = 13.9144, *p* < 0.001, ChaoSimpson: F_5, 60_ = 12.4671, *p* < 0.001, ChaoEvenness: F_5, 60_ = 11.8723, *p* < 0.001), but not between both lakes.

Beta diversity of *Oomycota* was strongly influenced by the lakes (PERMANOVA: F_1, 60_ = 17.307, *p* < 0.001) and habitats (F_5, 60_ = 13.184, *p* < 0.001). The influence of lake and habitat type on the *Oomycota* community composition was not consistent but differed between lakes, or habitat types, respectively (lake: habitat interaction, F_5, 60_ = 11.026, *p* < 0.001), i.e., samples from the same habitat type were not necessarily more similar to one another when originating from different lakes, and the two lakes did not show the same patterns for all habitats (Fig. 3b, Supplementary Figure S3). Overall, the sampling sites exhibited distinct community compositions, with some notable patterns emerging. Terrestrial forest samples (soil and rotten leaves) were highly similar to each other, as were the freshwater samples, though not as pronounced. However, aquatic samples from Grosse Fuchskuhle grouped more closely with the terrestrial forest samples than those from Grosser Stechlinsee. Notably, rotten leaves (shoreline) from Grosse Fuchskuhle were very similar to rotten leaves (forest) from both lakes, and sediment (shoreline) samples from Grosse Fuchskuhle showed great similarity to soil (forest) samples from both lakes (Fig. 3b, Supplementary Figure S3). The tested factors collectively explained a substantial portion of the variability in community composition, with habitat and the lake: habitat interaction being the strongest drivers (R² = 0.08725 for lake, 0.33234 for habitat, 0.27792 for lake: habitat interaction).

### Shared and differentially abundant OTUs

The majority of obtained OTUs was shared between freshwater and terrestrial habitats from both lakes (228 out of 401 OTUs, Supplementary Figure S4) with very few exclusively detected in one specific lake or habitat type (aquatic or terrestrial). As an example, only two and one OTUs were exclusively detected in freshwater habitats of Grosser Stechlinsee and Grosse Fuchskuhle, respectively. The same was true for terrestrial habitats with zero OTUs exclusively detected in terrestrial habitats of any of the lakes. Notably, the freshwater habitats of both lakes shared a relatively high number of OTUs (47 of 401; 12%) that were completely absent from the terrestrial samples, while only one single OTU was found exclusively in the terrestrial habitats.

Taxa that have been historically reported to be mostly terrestrial (e.g., *Globisporangium*, *Phytophthora*, *Pythium*) were indeed more common in the terrestrial habitats (Fig. 3c), however, especially *Pythium* and *Globisporangium* were sometimes the dominant genera in freshwater habitats (e.g., in surface water (pelagic zone) from Grosse Fuchskuhle, or in rotten leaves (shoreline) of Grosser Stechlinsee (Supplementary Figure S1)). *Aphanomyces* and *Saprolegnia* were neither more abundant in freshwater nor in terrestrial habitats. Notably, both *Saprolegniomycetes* and *Peronosporomycetes* contained genera that were more abundant in terrestrial or freshwater habitats. For instance, in *Saprolegniomycetes*,* Apodachlya*, *Leptolegnia*, and *Pythiopsis* were more common in the terrestrial samples, whereas *Achlya* and *Blastulidium* were more abundant in aquatic environments. Similarly, in *Peronosporomycetes*, some genera such as *Halophytophtora* and the previously mentioned *Globisporangium*, *Phytophthora*, and *Pythium* were more prevalent in terrestrial samples, while genera like *Peronospora* and *Phytiogeton* were more abundant in aquatic samples (Fig. 3c).

### Overall diversity from isolation techniques

**Fig. 4:**
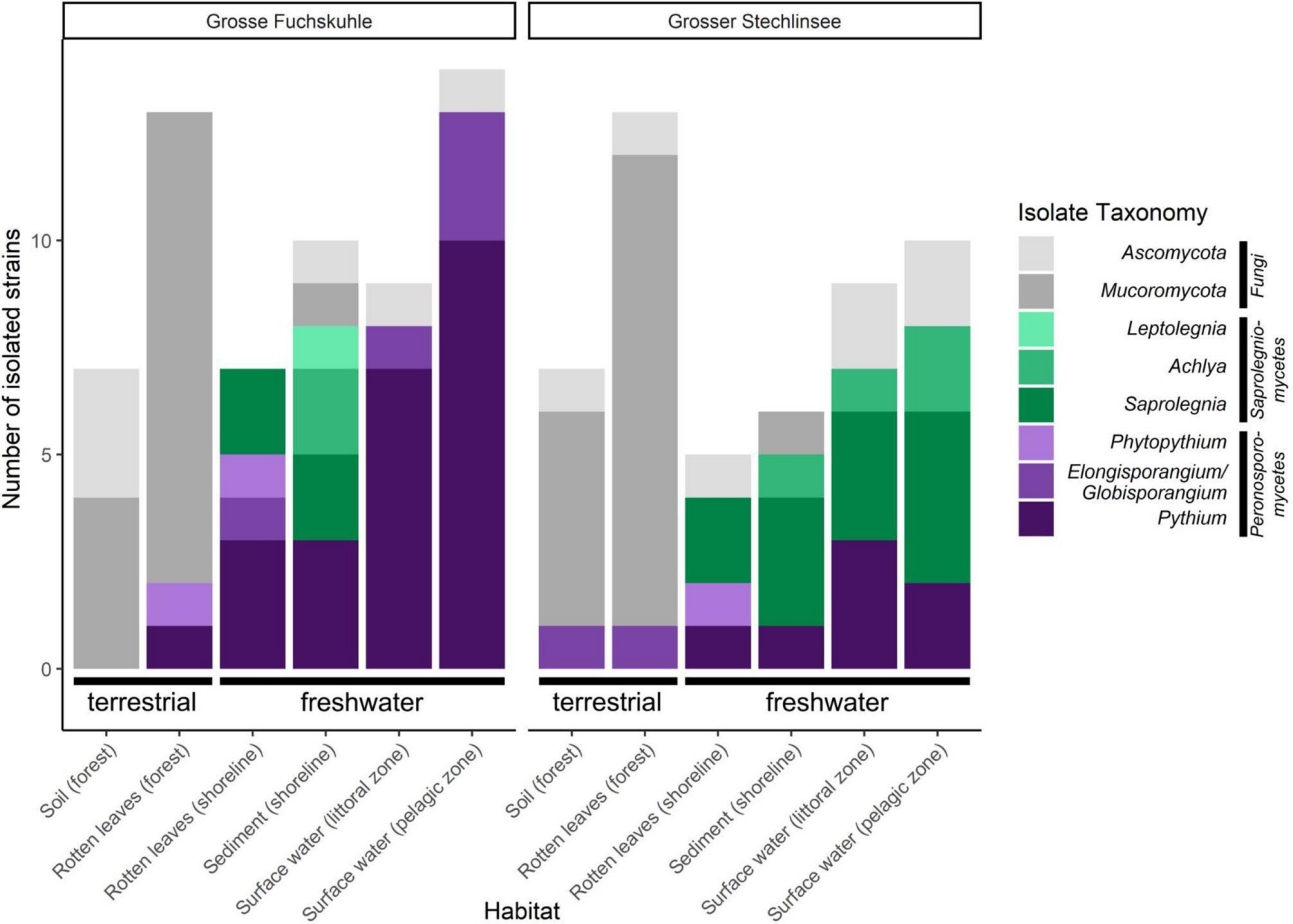
Diversity of isolated strains from six habitats of lakes Grosse Fuchskuhle and Grosser Stechlinsee (NE Germany) using selective culture-based baiting techniques for *Saprolegniales* and *Peronosporales*.

**Fig. 5:**
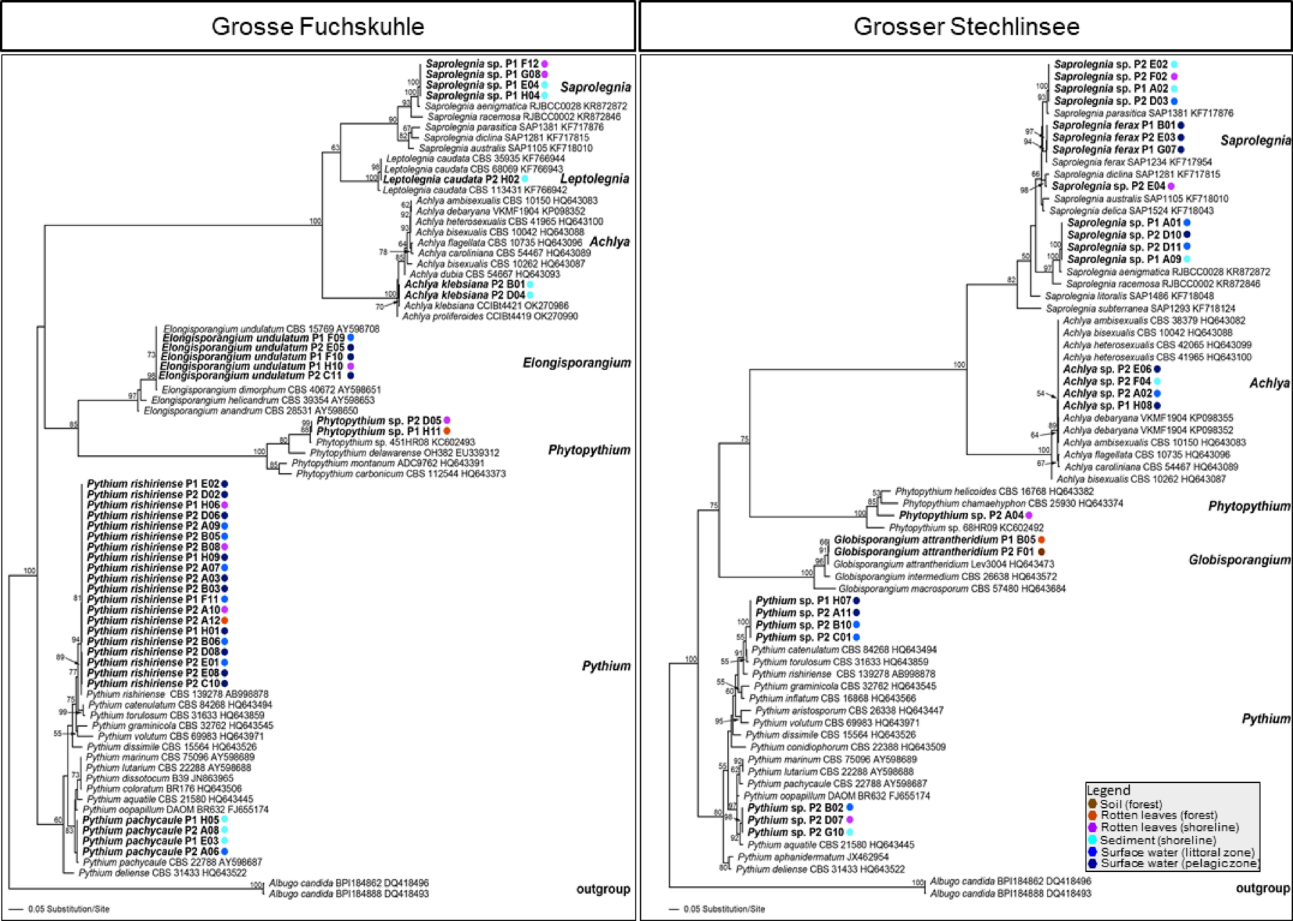
Phylograms of the best ML trees revealed by RAxML from an analysis of the internal transcribed spacers 1 and 2, and 5.8 S gene of rDNA sequences for *Saprolegniales* and *Peronosporales* strains isolated from Grosse Fuchskuhle and Grosser Stechlinsee using selective culture-based baiting techniques for each order.

In total, we isolated 110 strains from lakes Grosse Fuchskuhle (60) and Grosser Stechlinsee (50) using *Saprolegniales*- and *Peronosporales*-specific isolation methods. The success rate of *Oomycota* isolation was 59% as nearly 41% of isolated strains belonged to either *Ascomycota* and *Mucoromycota* (fungi), mostly isolated from either soil (forest) or rotten leaves (forest) habitats adjacent to both lakes (Fig. 4). The highest number of isolated *Oomycota* strains were associated with surface water (pelagic and littoral zones) and sediment (shoreline) in both lakes, respectively. Moreover, *Pythium sensu lato* and *Saprolegnia* were the most frequent and diverse *Oomycota* strains as they were isolated from all habitats and phylogenetically associated with different species based on ITS sequences (Fig. 5). In Grosse Fuchskuhle, strains were grouped in seven clades associated with *Saprolegnia aenigmatica*, *Leptolegnia caudata*, *Achlya klebsiana*, *Elongisporangium undulatum*, *Phytopythium* sp., *Pythium rishiriense*, and *P. pachycaule* (Fig. 5). Additionally, strains from Grosser Stechlinsee were closely related to four, one, and four species in *Saprolegnia*, *Achlya*, and *Pythium sensu lato* clades, respectively (Fig. 5).

In most cases, strains that were grouped in one clade (= identical based on their ITS sequences) had been isolated from two or three types of freshwater habitats. For instance, in Grosse Fuchskuhle, *Elongisporangium* sp. P1 F09, P2 E05, and P1 H10 were isolated from both surface water (littoral and pelagic) and rotten leaves (shoreline). The same is true for Grosser Stechlinsee, where *Pythium* sp. P2 D07, P2 G10, and P2 B02 had been isolated from rotten leaves and sediment (shoreline), and surface water (littoral zone) (Fig. 5).

## Discussion

While previous research using culture-based baiting methods has revealed the presence of *Saprolegniomycetes* and *Peronosporomycetes* in both freshwater and terrestrial ecosystems, it often lacked a comparative framework to examine their co-occurrence and relative distributions in both habitats. Our study addressed this gap using a metabarcoding approach, enabling a systematic analysis of the *Oomycota* community across freshwater and terrestrial ecosystems. By complementing metabarcoding with classical isolation techniques, we revealed patterns of habitat overlap and specificity that were previously obscured by methodological biases. We observed a striking pattern of low sequence similarity to reference database matches among *Saprolegniomycetes* OTUs in terrestrial ecosystems and *Peronosporomycetes* OTUs in freshwater habitats, suggesting the presence of numerous potentially undescribed taxa and highlighting their underexplored diversity.

Genera such as *Phytophthora* and *Pythium* (*Peronosporomycetes*) are commonly recognized as plant pathogens, whereas *Achlya*, *Aphanomyces*, and *Saprolegnia* (*Saprolegniomycetes*) are known to cause diseases in amphibians, crayfish, and fish^21,22^. Nevertheless, the above-mentioned separation is gradually being faded, particularly in the light of high throughput techniques such as metabarcoding. Supported by several metabarcoding-based studies, it has been shown that *Saprolegniomycetes* and *Peronosporomycetes* normally co-occur in soil^18^ and, to a much lesser extent, in water environments^20^. Fiore-Donno and Bonkowski (2021)^23^ deployed the metabarcoding approach used in this study to understand the distribution of *Oomycota* in temperate grasslands and forests. Up to 21% of their detected OTUs could be associated with *Saprolegniomycetes*. Our results showed a similar co-occurrence pattern in forest soil as well as freshwater habitats. Additionally, although we successfully isolated both *Saprolegniomycetes* and *Peronosporomycetes* strains using culture-based techniques, the limitations of these methods must be acknowledged. Firstly, culture-based techniques showed low efficiency, particularly in terrestrial habitats (soil and rotten leaves) where most of the isolated strains were either *Ascomycota* or *Mucoromycota* (fungi). Secondly, these methods distorted the overall picture of diversity as several *Oomycota* genera such as *Apodachlya*, *Phytophthora*, *Pythiopsis*, *Lagena*, and *Lagenidium* only detected by the metabarcoding approach. Finally, although hemp seeds and grass are common types of bait for the isolation of *Saprolegniomycetes* and *Peronosporomycetes* strains, they could introduce further biases as they might not be effective in the isolation of some genera.

A deeper understanding of *Oomycota* diversity across habitats raises important questions about the ecological roles of common as well as rare taxa. Our findings revealed that although *Pythium sensu lato* significantly contributes to the diversity of the community even in freshwater habitats, very little is known about their potential function(s). It could be either associated with fish and/or other aquatic plant diseases^24–27^, exploit freshwater environments as a transmission route for infection of terrestrial plants and further geographic distribution^28,29^, or simply act as litter decomposers.

Additionally, the significant presence of *Lagena* OTUs in the Grosser Stechlinsee freshwater habitats challenged the current notion of this genus as a soil inhabitant^23,30^ and/or causal agent of brown root rot in wheat and related grasses^31–33^. Our results can be explained by the fact that *Lagena* is also reported to be widely present in the roots of *Phragmites australis*^34^ which is, interestingly, the main shoreline vegetation of Grosser Stechlinsee. More recently, *Lagena* has been detected from a stream in Iceland with parasitoid nature against diatoms which is phylogenetically separated from the plant-associated clade^35^. Whether our *Lagena* OTUs are associated with the plant- or diatom-infecting clades remains to be answered in the future.

Moreover, the detection of *Saprolegnia*, a commonly known fish and amphibian pathogen^36,37^ from the soil samples of Grosse Fuchskuhle supports classic taxonomy-based studies where it has been reported from soil environments^38,39^. However, their possible ecological contribution to soil environments remains unanswered. One could argue that *Saprolegnia* might have undergone specific evolutionary adaptations and ecological versatility, allowing it to explore both land and water and acquire certain lifestyles. This argument is supported by the evidence from *Saprolegnia*’s close evolutionary relative, *Aphanomyces*, which comprises certain taxa as plant pathogens and others as crayfish and fish pathogens^4–9^.

It is not surprising that the observed diversity of the *Oomycota* community in our study is strongly influenced by the lake type, as Grosse Fuchskuhle and Grosser Stechlinsee differed markedly in their trophic status^40,41^. In particular, Grosse Fuchskuhle is a humic matter-rich lake which is influential in determining its biodiversity and ecological dynamics. For example, Perkins et al. (2019)^42^ and Allgaier and Grossart (2006)^43^ reported significant differences in fungal and bacterial community and diversity between the two lakes, mainly driven from their distinctive dissolved organic matter (DOM) composition. Although our results showed robust differences between the *Oomycota* community of both lakes, the underlying mechanisms driving this connection remain unclear and warrant further investigation. There are culture-based studies showing the possible impact of DOM, including humic substances, on sporulation^44^, vegetative growth^45^, and ability to degrade/transform refractory organic matter by some members of *Oomycota*^46,47^. Further studies incorporating experimental approaches and high-resolution molecular techniques are necessary to unravel the specific interactions between *Oomycota* and DOM components, as well as their broader implications for ecosystem processes in humic matter-rich versus clear-water lakes.

Finally, we need to acknowledge the limitations of our study to guide future research directions. Firstly, since the metabarcoding approach does not take viability into account^48^, we cannot be sure whether all detected OTUs are active and contributing to the ecology of their habitats. It is plausible that some detected OTUs are dead/dormant propagules of *Oomycota* washed into the freshwater habitats or transported passively to the forest. Culture-based techniques can compensate for this limitation to some extent but can be implemented only on a small scale and work on abundant, fast-growing *Oomycota*. Therefore, the metabarcoding approach should be complemented by methods such as metatranscriptomics^49^ in which functions of *Oomycota* can be investigated within their residing habitat.

Additionally, a comparison between the sequences of the isolates vs. the metabarcoding OTUs showed that some of the isolated strains do not have a proper match from our metabarcoding OTU database (Supplementary Material 2). This observation is partially explained by the fact that we used different sets of primers for both techniques, resulting in the amplification of different fragments that do not always match. Furthermore, it could be associated with the biases of culture-dependent techniques, as stated above, or due to amplification biases during the metabarcoding resulting in some OTUs which have been excluded during filtering steps due to low abundance in the dataset.

Besides, although we have included six different habitats along the aquatic-terrestrial ecotones of two distinct lakes in our study, the complexity of lake ecosystems may not be fully captured by our results. For instance, we exclusively collected floating rotten leaves from *Fagus sylvatica*, the dominant vegetation in the surrounding terrestrial environments of both lakes. Therefore, we might have encountered a different species composition of *Oomycota* if we collected leaves from other plant species. Moreover, one could argue that the diversity of *Oomycota* might experience successional shifts^50,51^, with certain OTUs colonizing less-decomposed leaves and others dominating as decomposition progresses. Accordingly, the *Oomycota* community is likely driven by seasonal changes, a factor not addressed in our study. Additionally, our results indicate that the level of eutrophication likely plays a role in influencing the community in freshwater, further highlighting the importance of environmental conditions. To address these limitations, we recommend further spatiotemporal studies^52,53^ to understand how the *Oomycota* community interacts with biotic and/or abiotic factors^54^ in their habitats over time.

## Conclusion

This study demonstrated that the *Oomycota* community is more diverse and widespread across interconnected freshwater and terrestrial habitats than previously assumed. Metabarcoding revealed a broad taxonomic range, including traditionally “aquatic” groups in soil and “terrestrial” groups in freshwater environments, while culture-based approaches highlighted certain biases and missed taxa. Environmental conditions, such as dissolved organic matter, lake type, and habitat characteristics, influenced the composition and distribution of *Oomycota*, resulting in a distinct community for each habitat, while still displaying a substantial ecological overlap between terrestrial and aquatic ecosystems. These findings challenge long-standing notions about *Oomycota* habitat preferences and underscore the importance of employing integrative techniques and broader sampling efforts to fully understand their ecological roles and environmental drivers.

## Methods

### Study sites and sampling design

We compared the *Oomycota* community of two contrasting lakes with distinctive nutrient availability differences, the oligo-/mesotrophic lake ‘Grosser Stechlinsee’ (53° 9’ 7.301” N, 13° 1’ 23.894” E) and the dystrophic lake ‘Grosse Fuchskuhle’ (53° 6’ 19.674” N, 12° 59’ 5.626” E) in northeast Germany plus their surrounding terrestrial habitats. Samples were collected in January 2023 from six different habitats, including surface water from pelagic zones and littoral, homogenized sediment (5 cm depth) from the shoreline, floating rotten leaves (*Fagus sylvatica* L) from the shoreline, rotten leaves from the forest floor and mineral soil of the forest floor (5 cm depth). Samples from lawn mineral soil (5 cm depth, 53° 08’ 29.1” N 13° 01’ 49.04” E) were used as positive controls, but not further analyzed. Six replicates were collected per sampling site. Surface water (littoral and pelagic), sediment (shoreline), and rotten leaves (shoreline) represented freshwater habitats while terrestrial habitats were represented by rotten leaves and mineral soil from forest.

### DNA extraction, amplification, and sequencing for the metabarcoding approach

Water samples were passed through 5.0 μm cyclopore track etched membranes (Cytiva, US) with reusable filter units (Thermo Fisher Scientific, US) connected to a portable 2 l single-stage vacuum pump (Value, Poland). The membranes were placed in 2 ml sterilized safe-lock tubes. Additionally, three grams of sediment or soil, and single rotten leaves (cut in 25 pieces, 1 cm x 1 cm in size) were put in separate 2 ml sterilized safe-lock tubes. All tubes were kept at − 80 °C until DNA extraction.

DNA extraction was conducted as follows^55^: Firstly, one full lab scoop of zirconium beads with three sizes (0.1, 0.7, and 1 mm) were equally added to 2 ml sterilized safe-lock tubes containing folded 5.0 μm cyclopore track etched membranes, sediment, rotten leaves, or soil. Then, 650 µl of 2X CTAB-Buffer (1 g 2% CTAB, 4.09 g 1.4 M NaCl, 0.29225 g 20 mM EDTA, and 0.788 g 100 mM Tris HCl filled with Diethylpyrocarbonat-water to 50 ml pH 8.0) was added, followed by 65 µl 10% Sodium dodecyl sulfate (SDS), 65 µl 10% N-Lauroylsarcosine, and 10.4 µl Proteinase K (20 mg/ml). Tubes were kept in the thermomixer for one hour at 60 °C. 650 µl Phenol-Chloroform-Isoamyl Alcohol (25:24:1) was added to each tube and placed in the FastPrep (MP Biomedicals) under the recommended setting. Tubes were then centrifuged for 10 min at 14,000 x g at 4 °C, followed by transferring 650 µl of the upper phase into new tubes. 650 µl Chloroform-Isoamylalkohol was added to each new tube and centrifuged once again (14000 x g at 4 °C). The upper phase was transferred to new tubes containing 2 Vol. (1300 µl) PEG/NaCl (4.7 g 1.6 M NaCl and 15 g 30% PEG 6000 filled with 50 ml DEPC-water). Tubes were incubated at 4 °C for at least 12 h. Tubes were centrifuged for 60 min at 17,000 x g at 4 °C, and the supernatant was removed. The pellet was washed with 800 µl 70% ice-cold ethanol and centrifuged (17000 x g for 10 min at 4 °C). 70% ethanol was pipetted off from tubes completely. Finally, 50 µl 10 mM Tris was added to the tubes and incubated for one hour at 35 °C. The final products were stored at −20 °C.

DNA was diluted 1:30 in nuclease-free water before PCRs. The ITS1 region of *Oomycota* was amplified in a semi-nested metabarcoding approach using *Oomycota*-specific primers^23^. Two consecutive PCRs were performed: The first PCR was conducted with the primer pair S1777F (5′-GGTGAACCTGCGGAAGGA-3′) and 58SOomR (5′-TCTTCATCGDTGTGCGAGC-3′), and the second PCR with barcoded primers S1786StraF (5′-GCGGAAGGATCATTACCAC-3′) and barcoded 58SOomR^23^. Each sample was amplified with a unique combination of barcoded primers (double indexing) in the second PCR (Supplementary Material 3). One µl of purified DNA served as template for the first PCR, resulting in amplicons of which 1 µl was used for the second semi-nested PCR. Both PCR rounds were conducted with reagents in the following final concentrations: DreamTaq polymerase (Thermo Fisher Scientific, Dreieich, Germany) 0.01 units, Thermo Scientific DreamTaq Green Buffer, dNTPs 0.2 mM, and primers 1 mM. Both PCRs were conducted using the following PCR conditions: Initial denaturation at 95 °C for 2 min, 24 cycles (denaturation at 95 °C for 30 s, annealing at 58 °C for 30 s, elongation at 72 °C for 30 s), terminal extension at 72 °C for 5 min, and cooling at 15 °C. All PCRs were conducted in duplicates. Positive controls (DNA from grassland soil) as well as negative controls (water) were added to each PCR batch. After checking for successful amplification by gel electrophoresis, 12.5 µl of the two PCR products of each sample were pooled, purified, and normalized using the SequalPrep Normalization Plate Kit (Invitrogen GmbH, Karlsruhe, Germany) to obtain a concentration of 1–2 ng/µl per sample. All purified amplicons were then pooled, concentrated, and sequenced on a MiSeq platform (Illumina Inc., San Diego, CA, USA) at the Cologne Center for Genomics (Germany).

### Sequence processing

Reads were processed in mothur v.1.45.3^56^. Reads were assembled and demultiplexed according to their barcode sequences, not allowing any mismatches in the barcode and primer sequences, allowing a maximum of two mismatches, and no ambiguities in the sequences. Assembled sequences with an overlap < 170 bp were discarded. Merged contigs were demultiplexed and primer and tag sequences were trimmed. Remaining reads were clustered into operational taxonomic units (OTUs) by abundance-based greedy clustering (agc) with a similarity threshold of 97% using vsearch^57^. OTUs representing less than 0.005% of all reads (i.e., < 106 reads) were removed as they likely represent amplification or sequencing errors^58^. Sequences were blasted (Nucleotide BLAST, blastn) against a custom *Oomycota* database based on NCBI GenBank with an e-value of 1e^−10^. Only the best hit was retained, and non-*Oomycota* sequences were discarded. Sequences with an alignment to sequence length ratio of less than 0.7 were removed since aligning to a reference alignment and chimera detection often led to false eliminations of sequences due to the high variability in length of the ITS1 region. The quality-filtered OTU database was used for all subsequent statistical analyses.

The taxonomic assignment was manually curated. In particular, OTUs assigned to *Pythium* needed manual correction since the genus *Pythium* has undergone taxonomic changes during the last years^59^, and many sequences in the NCBI database are still falsely assigned to *Pythium*. Representative OTU sequences were manually re-blasted (blastn) against the full NCBI GenBank database for accurate genus-level identification, and classifications were updated to reflect current taxonomy. A rough assignment of ecological traits to the OTUs was performed on genus-level^23^.

### Statistical analyses

All data analyses were conducted in R version 4.1.1^60^. The respective R code is provided in the supplement (Supplementary Material 4). Sankey diagrams displaying the relative abundance at different taxonomic resolutions were calculated for each sampling site with the riverplot package^61^ using a custom function from Freudenthal et al. (2024)^62^. Additionally, pie charts displaying the relative abundance of orders were calculated using R base functions and ggplot2^63^.

The data was not rarefied, but instead extrapolated by using the iNEXT package^64^ for the calculation of alpha diversity indices. From the OTU tables, the extrapolated alpha diversity indices OTU richness, exponential Shannon index, and inverse Simpson index were calculated using iNEXT::ChaoRichness, iNEXT::ChaoShannon, and iNEXT::ChaoSimpson, respectively. Pielou’s evenness index was then calculated from extrapolated Shannon index and OTU richness. Differences in alpha diversity metrics were analyzed using Type I SS analysis of variance (ANOVA). A post-hoc Tukey’s honest significance test (0.05 significance level) was performed for pair-wise comparisons of the groups.

Differences in community structure between the sampling sites (beta diversity) were investigated by multivariate approaches. Total abundances of OTUs were converted to relative abundances and log(+ 1) transformed. Non-metric multidimensional scaling (NMDS) based on Bray-Curtis dissimilarities was performed using the vegan::metaMDS function^65^. Initial two-dimensional NMDS analysis yielded a stress value greater than 0.2, which indicates a poor fit and insufficient representation of the data in a reduced-dimensional space. To address this, we performed three-dimensional NMDS, which provided a better representation of the data. NMDS was visualized to depict patterns in community similarity/dissimilarity, focusing on the influence of the sampled habitats and lakes. Statistical testing was performed with permutational multivariate analysis of variance (PERMANOVA, vegan::adonis2, 9999 permutations, Bray-Curtis dissimilarities). The model was constructed in the same way as the ANOVA models.

Venn diagrams displaying the shared OTUs between lakes and aquatic/terrestrial habitats were constructed using the VennDiagram package^66^. Differential abundance analysis was conducted in DESeq2^67^ and displayed as a heat tree using metacoder^68^.

### Isolation of *Saprolegniales* and *Peronosporales* strains

Soil (3 g), sediment (3 g), and one rotten leaf (10 pieces from the same leaf used for metabarcoding, 1 cm x 1 cm in size) were transferred to separate 10 cm Petri dishes containing 15 ml of autoclaved lake water with 25 boiled hemp seeds (for *Saprolegniales*) or CMA medium (40 gl-1 ground cornmeal, 15 gl-1 agar) (for *Peronosporales*); both autoclaved lake water and CMA medium were amended with ampicillin and fluconazole (0.1 g/L each) to avoid fungal and/or bacterial contamination^69^. Petri dishes were sealed, incubated at room temperature, and daily checked for colonization. Upon colonization, mycelium was transferred to CMA agar with antibiotics. This step was repeated two more times until any visible fungal and/or bacterial contaminations were removed.

For single-spore isolation, five small blocks of CMA agar (1 cm x 1 cm) containing mycelia were transferred into a Petri dish containing autoclaved lake water inoculated with 25 boiled hemp seeds (for *Saprolegniales*) and boiled grass (for *Peronosporales*). Petri dishes were checked every 12 h for zoospore sporulation. Upon zoospore sporulation, 5 µl liquid was collected and transferred to a Petri dish containing CMA agar with antibiotics. The germination tube produced by one single zoospore was cut with a sterile needle and transferred to another Petri dish containing CMA agar with antibiotics. This Petri dish with the pure strain was kept at 4 °C for future use^46,70^.

For the isolation of *Oomycota* from water (littoral and pelagic zones), two cheesecloth bags containing 25 boiled hemp seeds (for *Saprolegniales*) or boiled grass (for *Peronosporales*) were attached to experimental platforms installed in the littoral and pelagic zones of the surface of water in both lakes. After five days, boiled hemp seeds were transferred to Petri dishes containing 10 ml of autoclaved lake water. The subsequent isolation procedure followed the process described above.

### DNA extraction, amplification, and sequencing for living ***Oomycota*** strains

DNA of *Oomycota* cultures was extracted using chelex method^71^ with some modifications: Briefly, strains’ mycelial mats were transferred to 1.5 mL Eppendorf tubes containing 150 µL of autoclaved 5% Chelex® 100 (BIO-RAD), incubated in a thermoblock at 100 °C for 10 minutes, vortexed for 10 seconds, and then returned to the thermoblock for another 10 minutes at 100 °C. Samples were subsequently centrifuged at 14,000 × g for 10 minutes at 4 °C. The resulting supernatants were stored at –20 °C until further use. The nuclear ITS-rDNA region was amplified in a Flexible PCR Thermocycler (Analytikjena, Germany) using ITS1/ITS4 primers^72^. The program for amplification was: 94 °C for 2 min of initial denaturation followed by 32 cycles of 94 °C for 15 s, 53 °C for 15 s, 72 °C for 30 s, and a final extension at 72 °C for 5 min. The resulting sequences were edited in BioEdit^73^ and submitted to GenBank (National Center for Biotechnology Information; http://www.ncbi.nlm.nih.gov).

### Phylogenetic analysis for living ***Oomycota*** strains

The generated sequences of our isolates were used as a query for BLASTn analyses in the NCBI database. Sequences linked to voucher cultures with high identity to the query were chosen for phylogenetic analysis, as well as additional sequences from possible relatives were added based on referencing literature in the database^74,75^. Alignments were produced with MAFFT v. 7.490 (http://mafft.cbrc.jp/alignment/server/^73^) and checked and refined using MEGA7^74^. After the exclusion of ambiguously aligned regions and long gaps, the final matrix was chosen. Maximum Likelihood (ML) analyses were performed with RAxML^78^ as implemented in raxmlGUI v. 1.3^76^ using the ML + rapid bootstrap setting and the GTRGAMMA substitution model with 1000 bootstrap replicates. A table summarizing all used strains, their corresponding sequences, and GenBank accession numbers can be found in Supplementary Table S1.

## Supplementary Information

Below is the link to the electronic supplementary material.

Supplementary Material 1

## Data Availability

The raw Illumina sequences are available in the European Nucleotide Archive (ENA) (https://www.ebi.ac.uk/ena/browser/home) under the accession number PRJEB77433 (secondary study accession: ERP161867). Sequences of isolated strains generated in this study (Supplementary Table S1) are available in NCBI GenBank (https://www.ncbi.nlm.nih.gov/genbank/) under the accession numbers PP995820-PP995845 and PP995782-PP995819. The datasets and R code are available in Supplementary Material 4. The results from Functional guilds are provided in Supplementary Table S2.
