# Supplementary material for: A glimpse into Oomycota diversity in freshwater lakes and adjacent forests using a metabarcoding approach
Source: Sci Rep. 2025 May 31;15:19124. doi: 10.1038/s41598-025-01727-3 (PMC12126517; doi:10.1038/s41598-025-01727-3)
Supplement: Supplementary file 1 — Supplementary Material 1 [file 41598_2025_1727_MOESM1_ESM.zip › Supplementary Figure S1.pptx]

## Slide 1
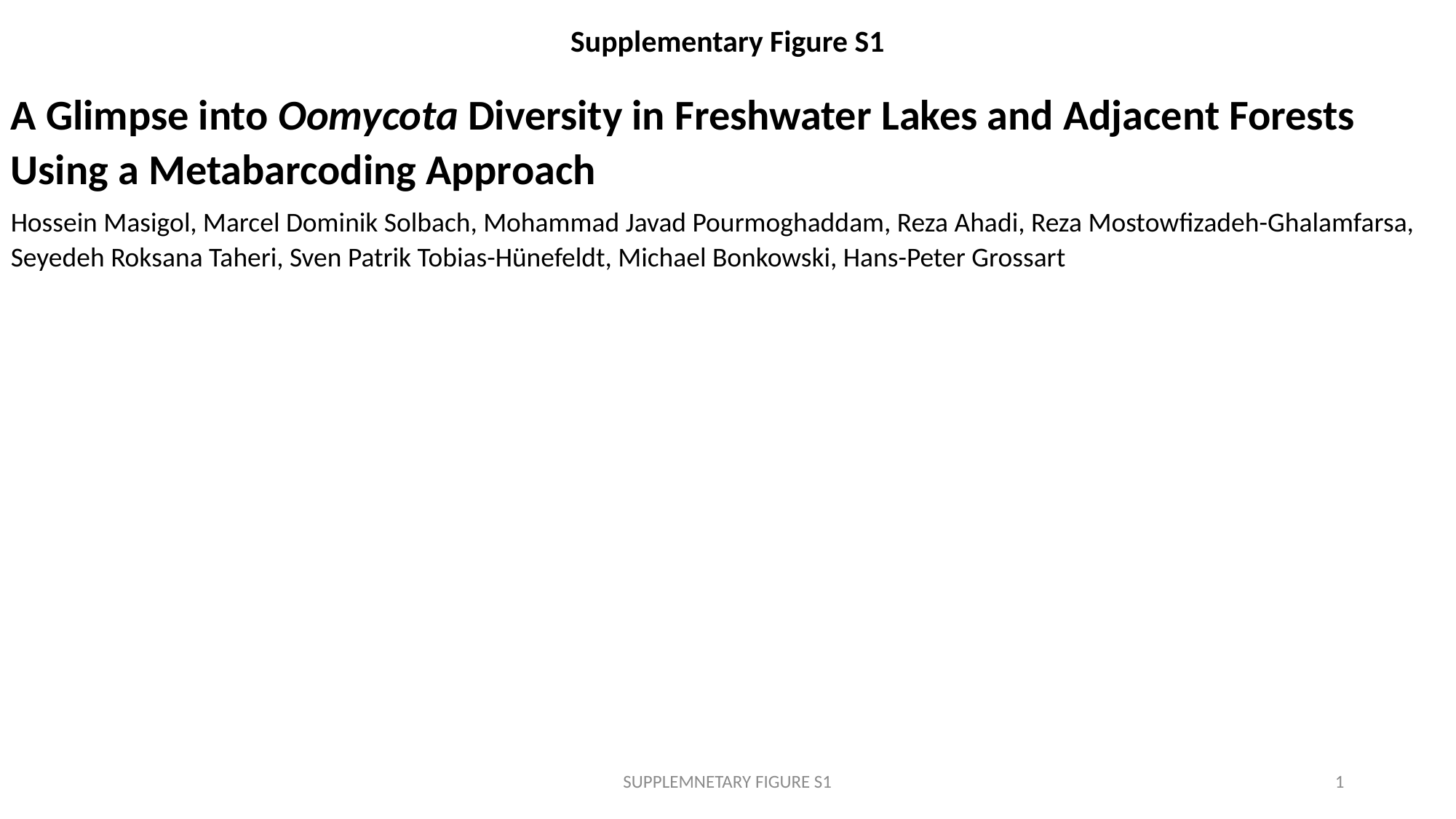

Supplementary Figure S1
A Glimpse into Oomycota Diversity in Freshwater Lakes and Adjacent Forests Using a Metabarcoding Approach
Hossein Masigol, Marcel Dominik Solbach, Mohammad Javad Pourmoghaddam, Reza Ahadi, Reza Mostowfizadeh-Ghalamfarsa, Seyedeh Roksana Taheri, Sven Patrik Tobias-Hünefeldt, Michael Bonkowski, Hans-Peter Grossart
SUPPLEMNETARY FIGURE S1
1

## Slide 2
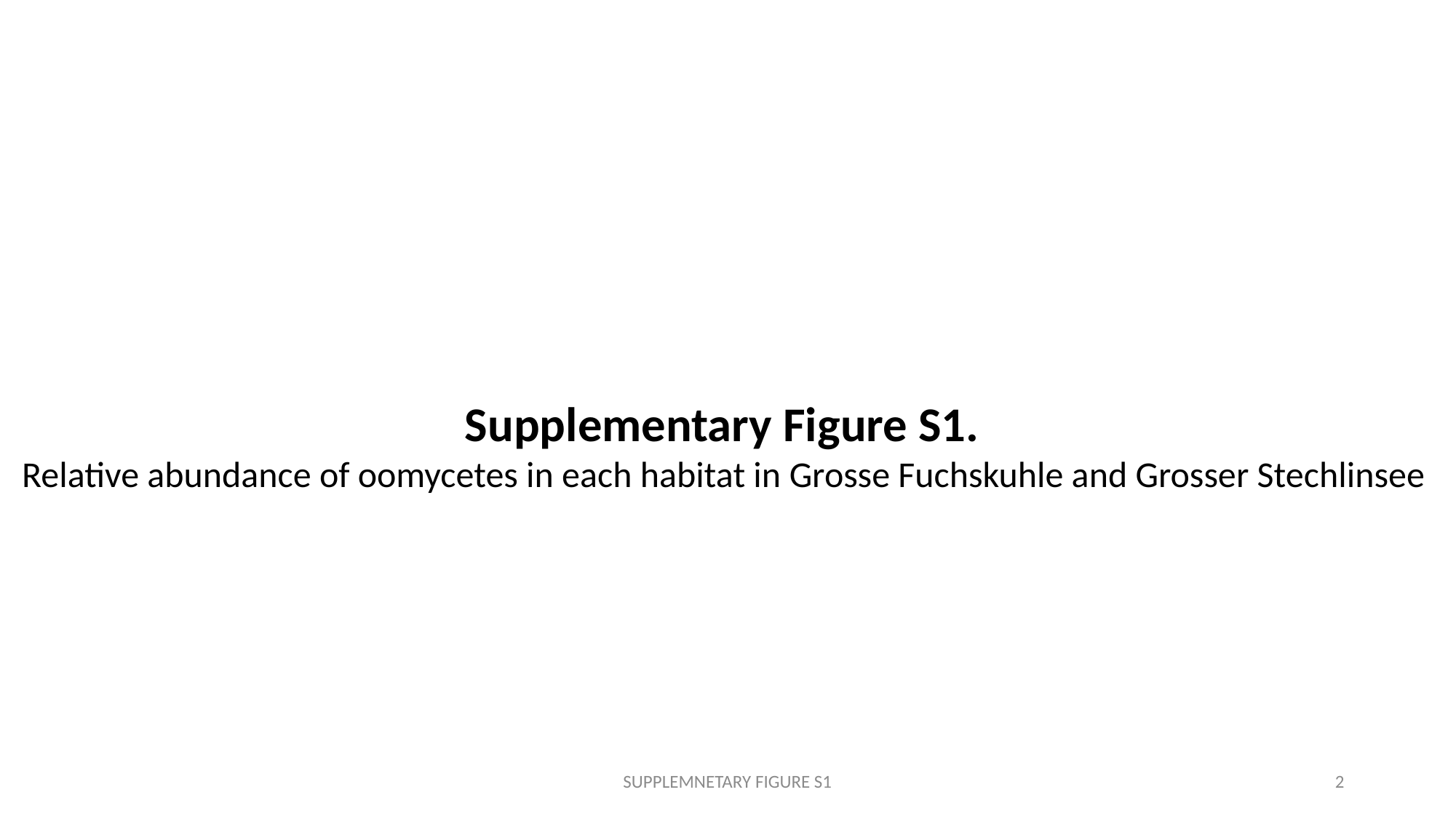

Supplementary Figure S1.
Relative abundance of oomycetes in each habitat in Grosse Fuchskuhle and Grosser Stechlinsee
SUPPLEMNETARY FIGURE S1
2

## Slide 3
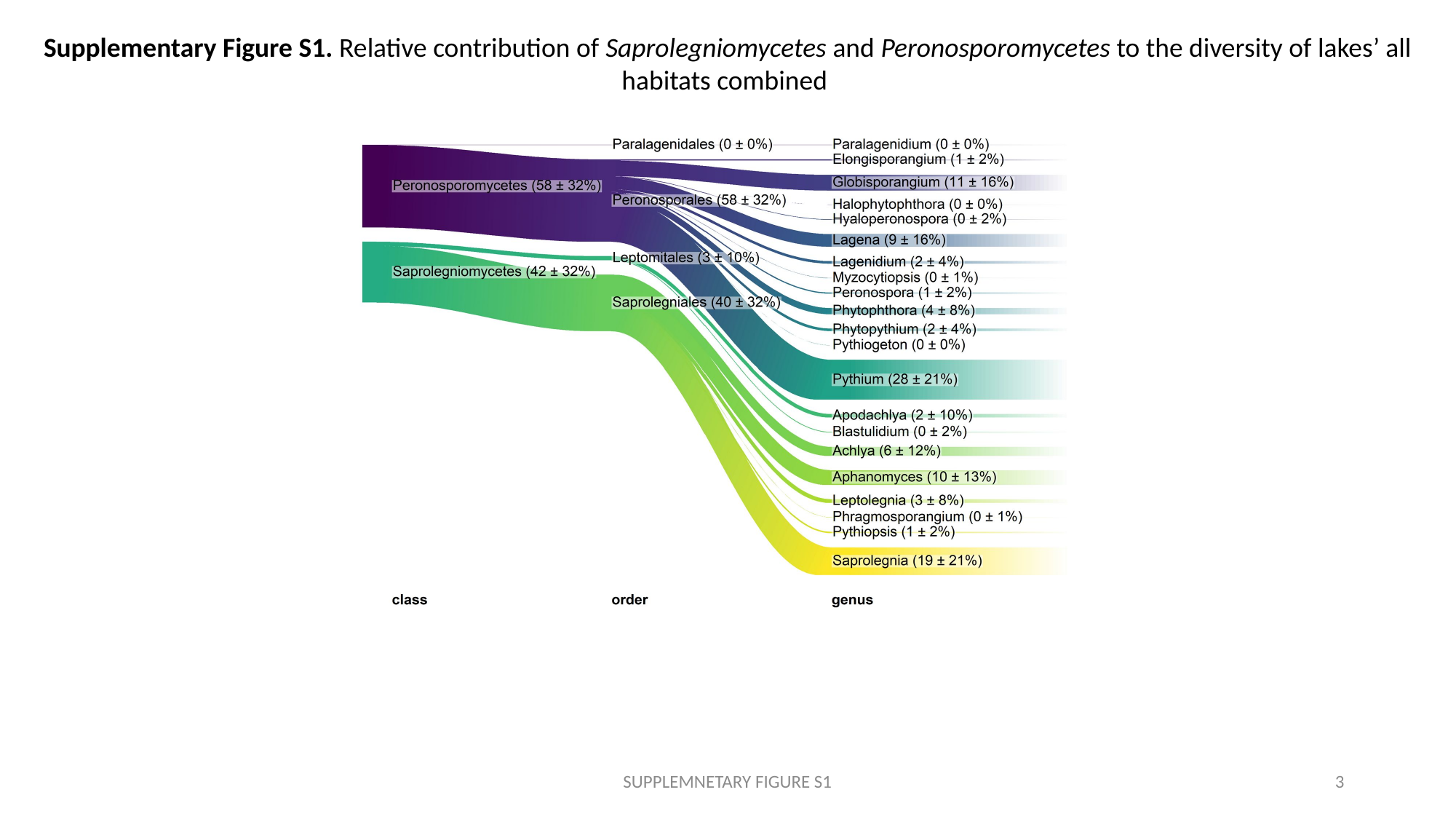

Supplementary Figure S1. Relative contribution of Saprolegniomycetes and Peronosporomycetes to the diversity of lakes’ all habitats combined
SUPPLEMNETARY FIGURE S1
3

## Slide 4
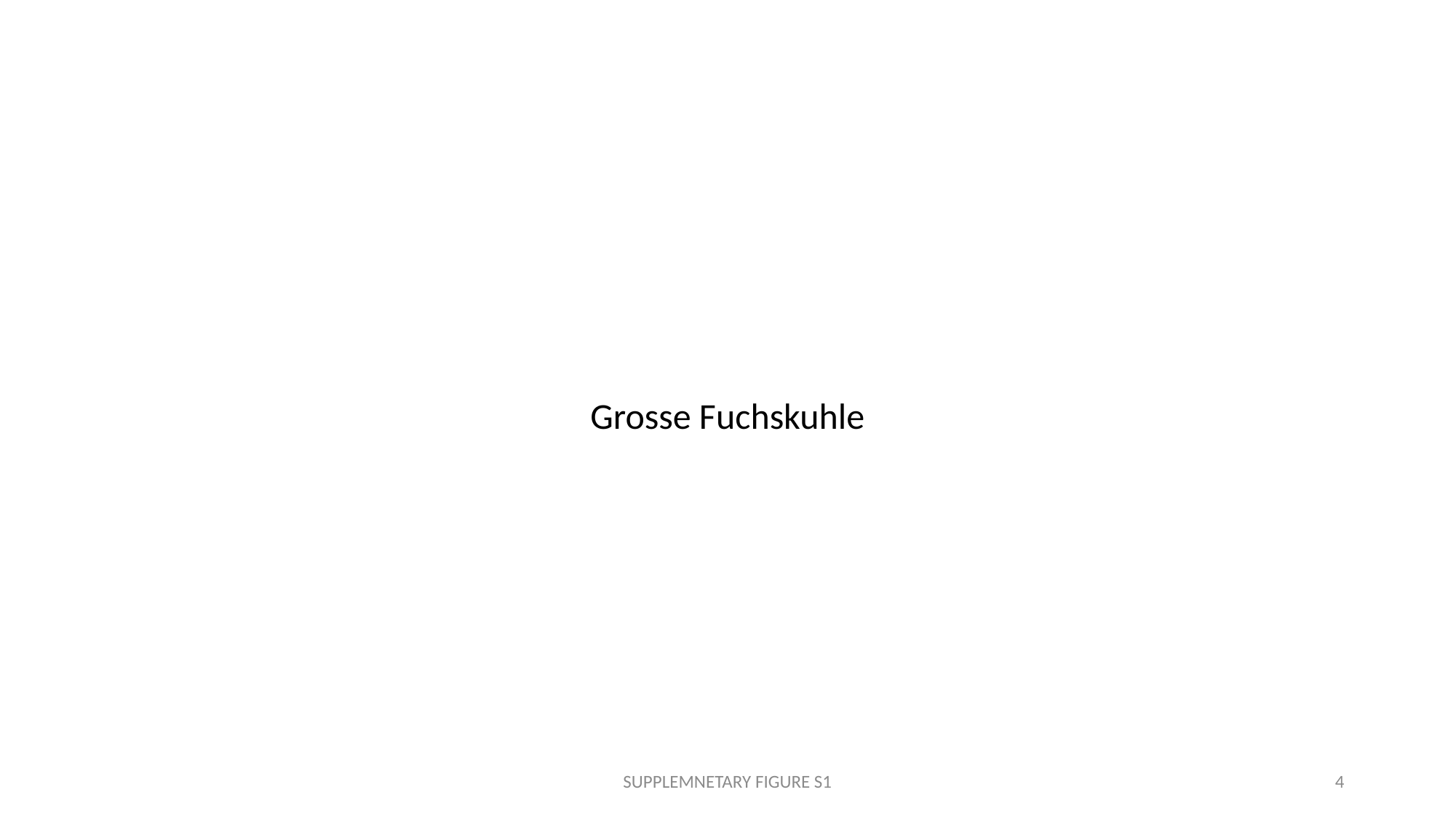

Grosse Fuchskuhle
SUPPLEMNETARY FIGURE S1
4

## Slide 5
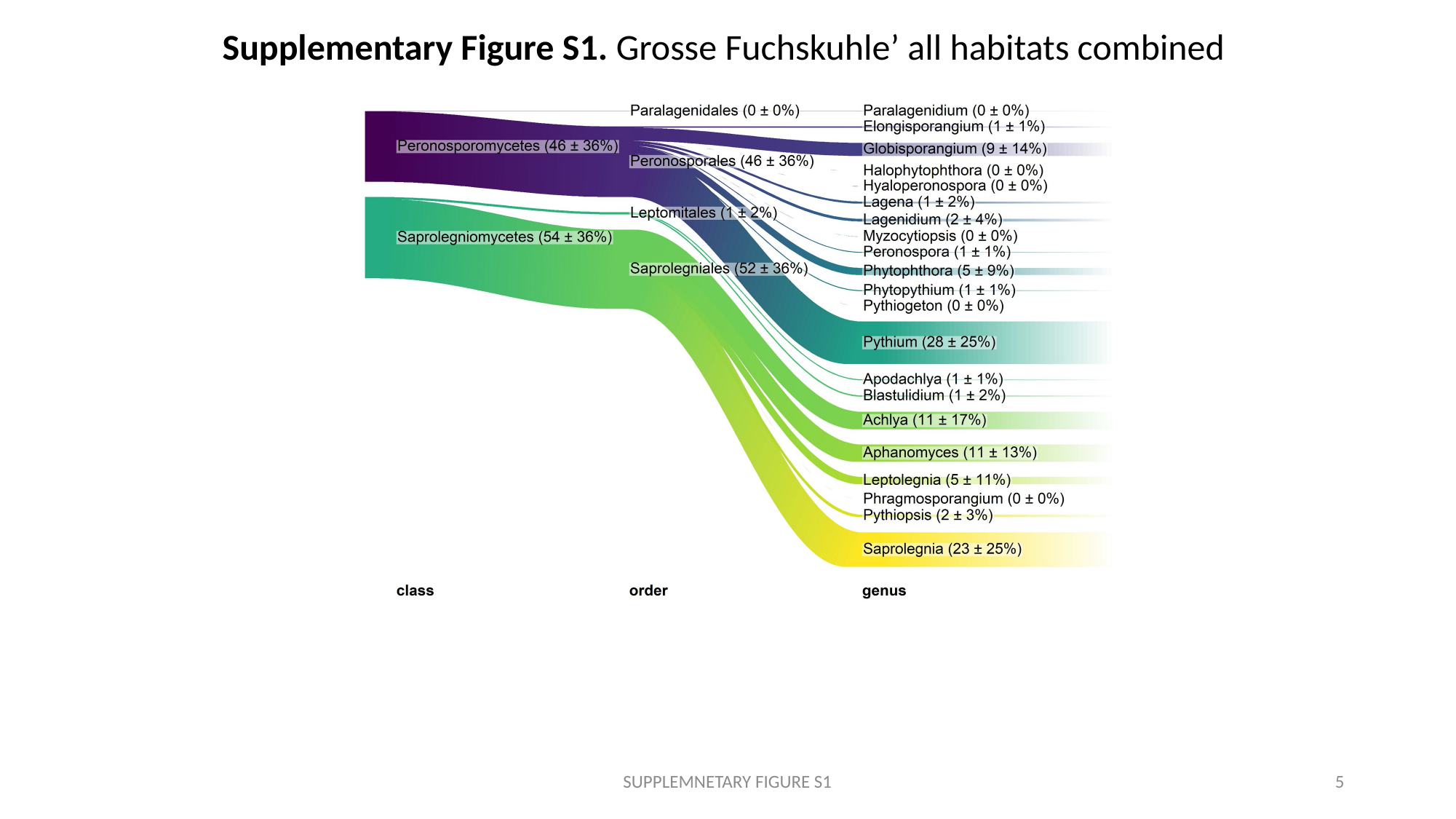

Supplementary Figure S1. Grosse Fuchskuhle’ all habitats combined
SUPPLEMNETARY FIGURE S1
5

## Slide 6
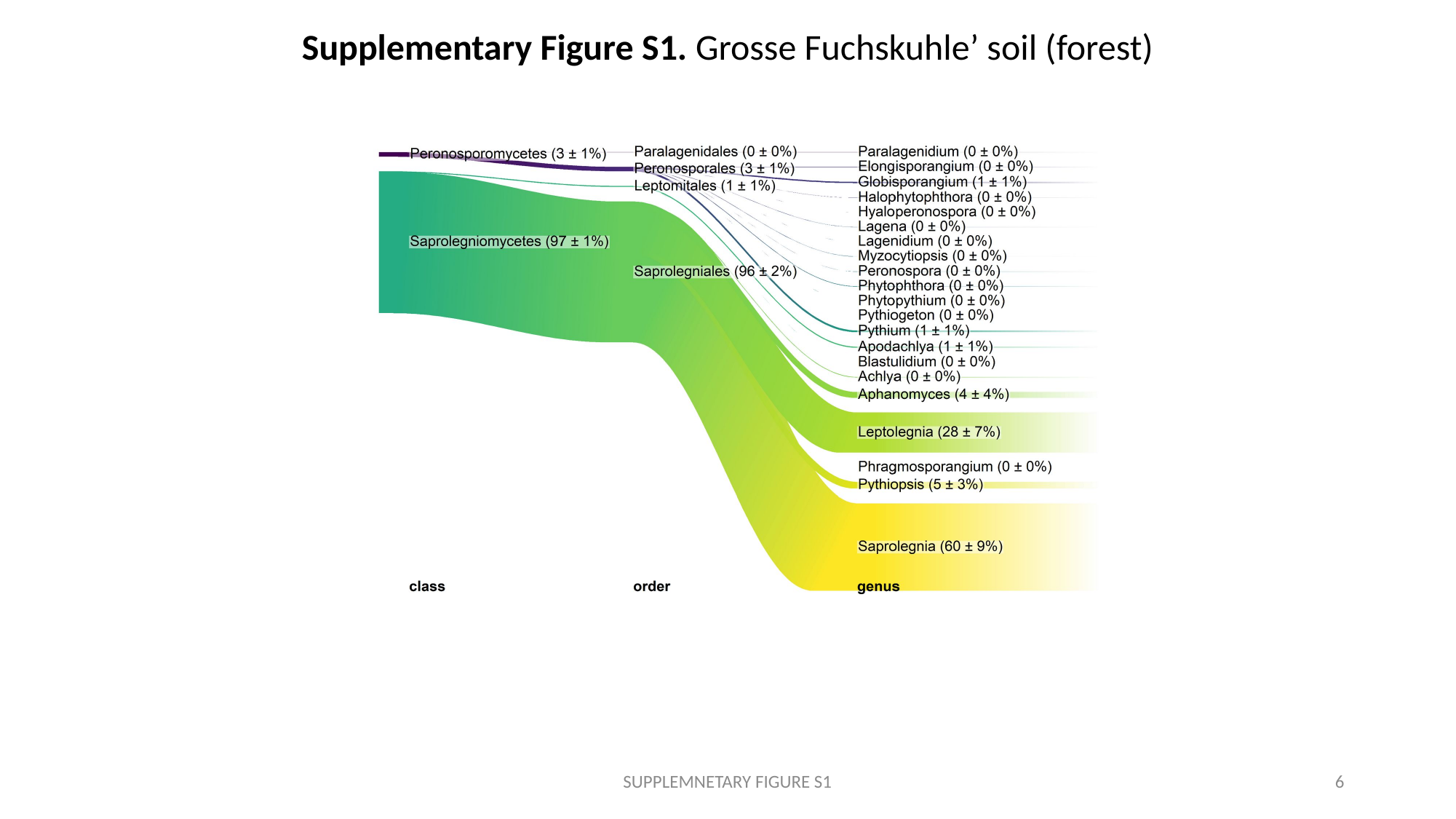

Supplementary Figure S1. Grosse Fuchskuhle’ soil (forest)
SUPPLEMNETARY FIGURE S1
6

## Slide 7
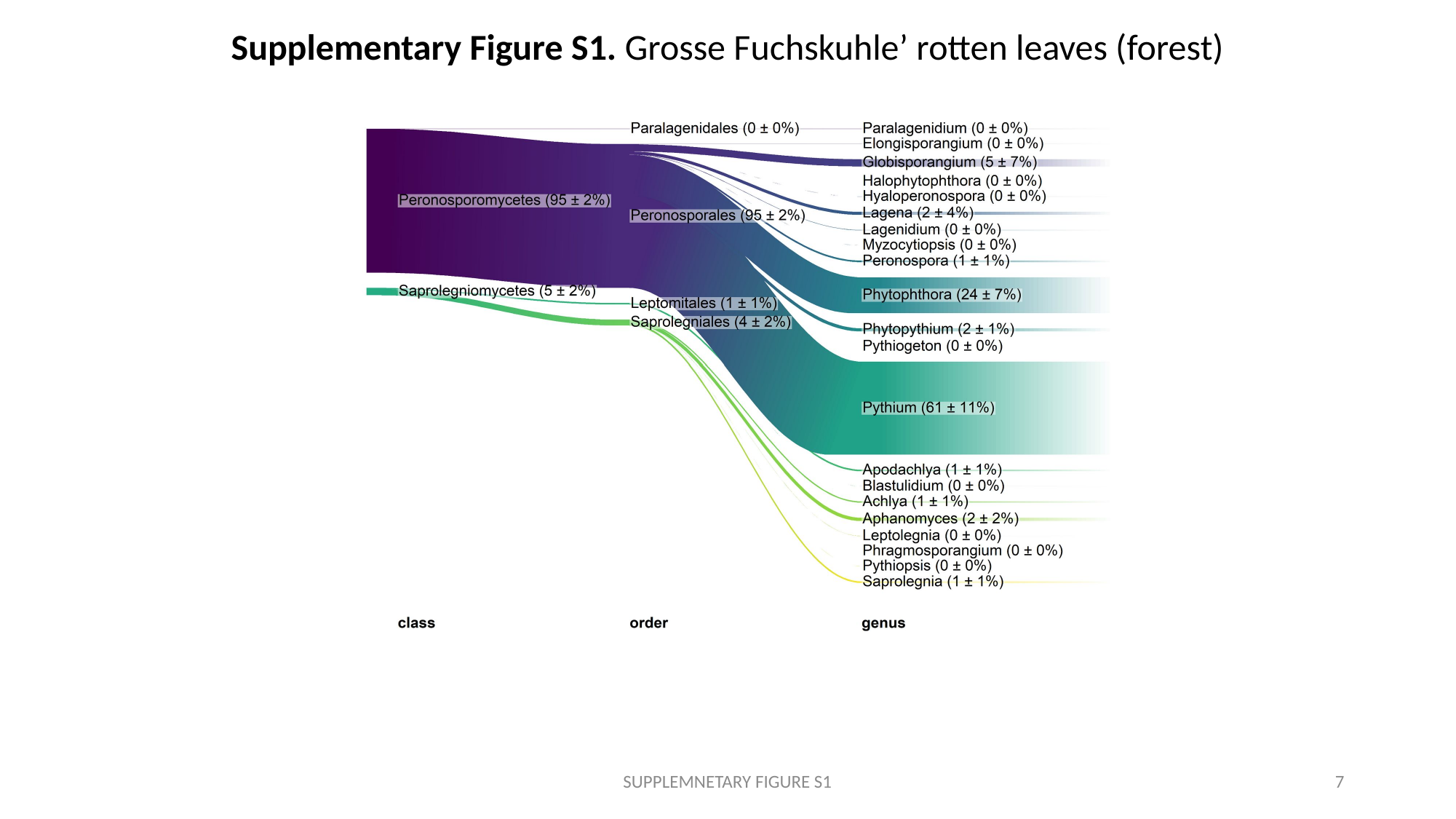

Supplementary Figure S1. Grosse Fuchskuhle’ rotten leaves (forest)
SUPPLEMNETARY FIGURE S1
7

## Slide 8
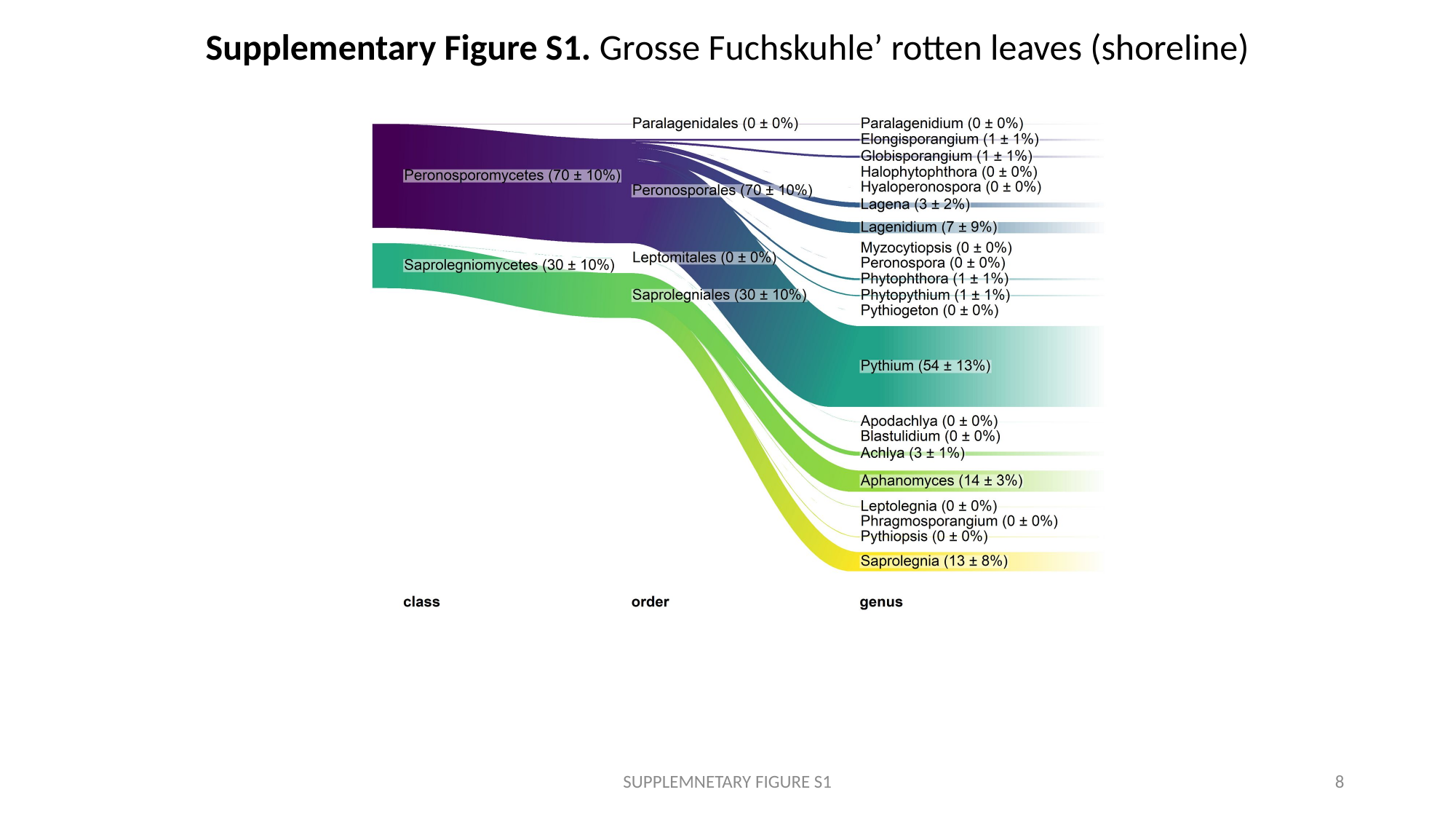

Supplementary Figure S1. Grosse Fuchskuhle’ rotten leaves (shoreline)
SUPPLEMNETARY FIGURE S1
8

## Slide 9
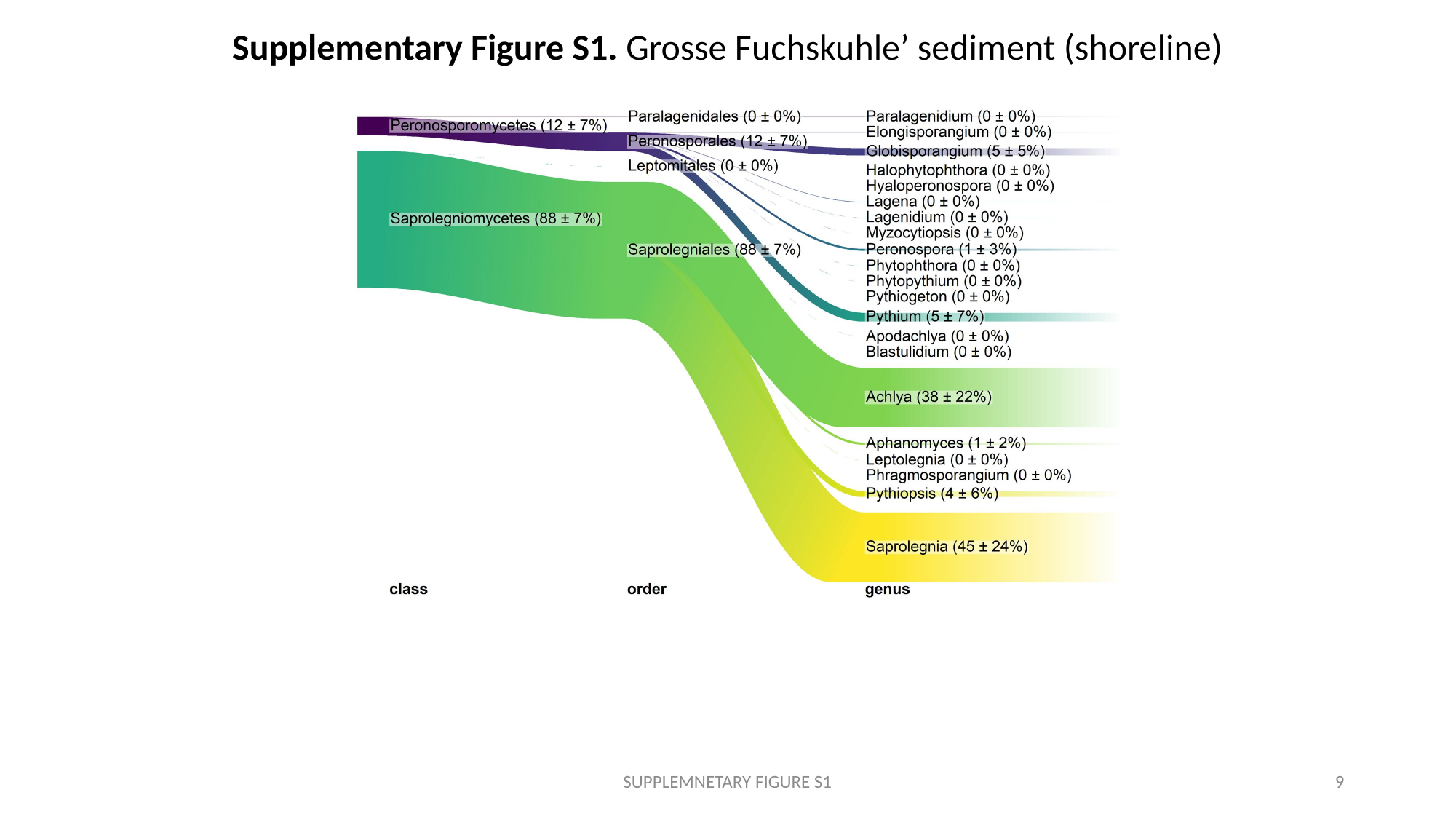

Supplementary Figure S1. Grosse Fuchskuhle’ sediment (shoreline)
SUPPLEMNETARY FIGURE S1
9

## Slide 10
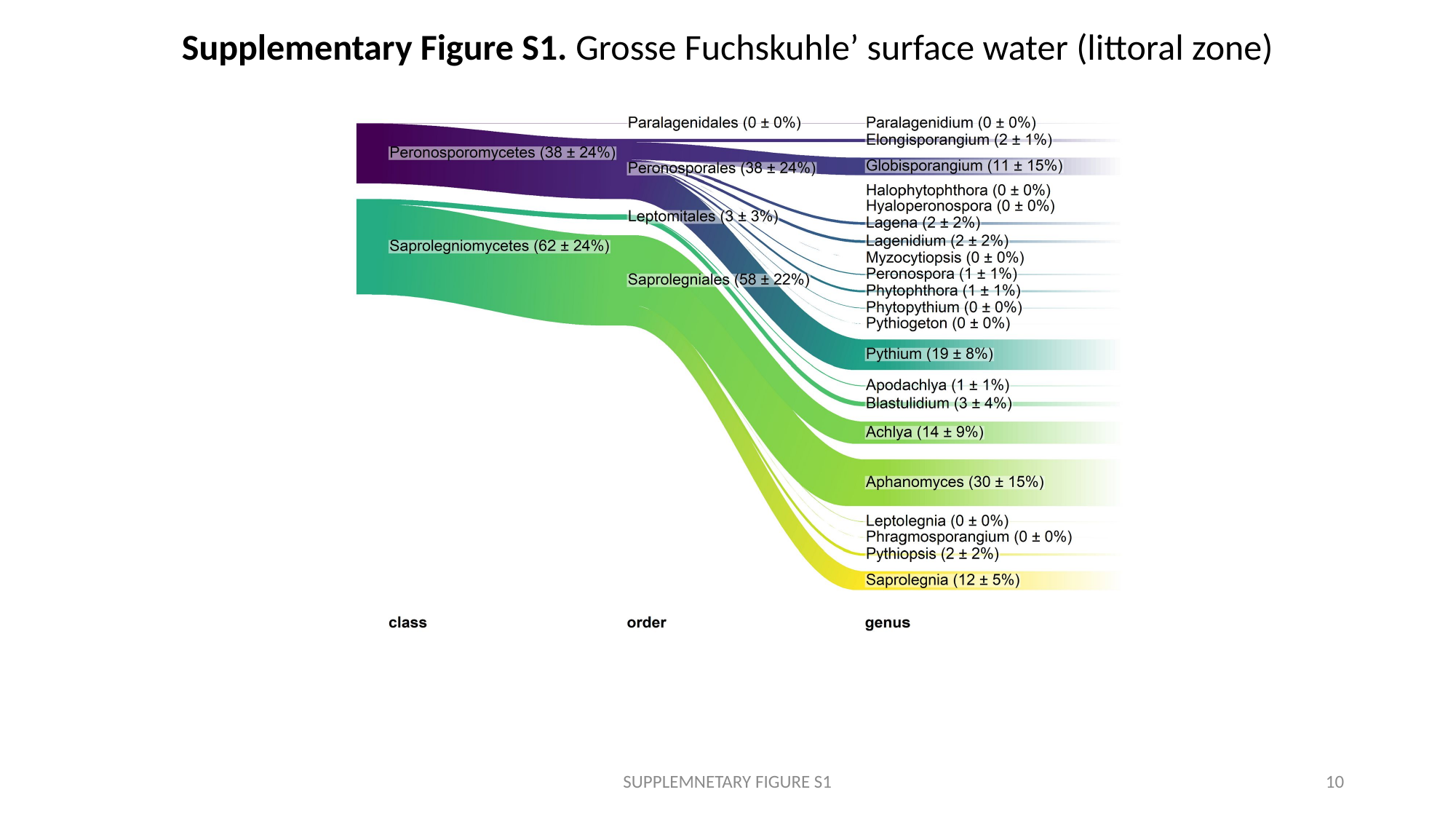

Supplementary Figure S1. Grosse Fuchskuhle’ surface water (littoral zone)
SUPPLEMNETARY FIGURE S1
10

## Slide 11
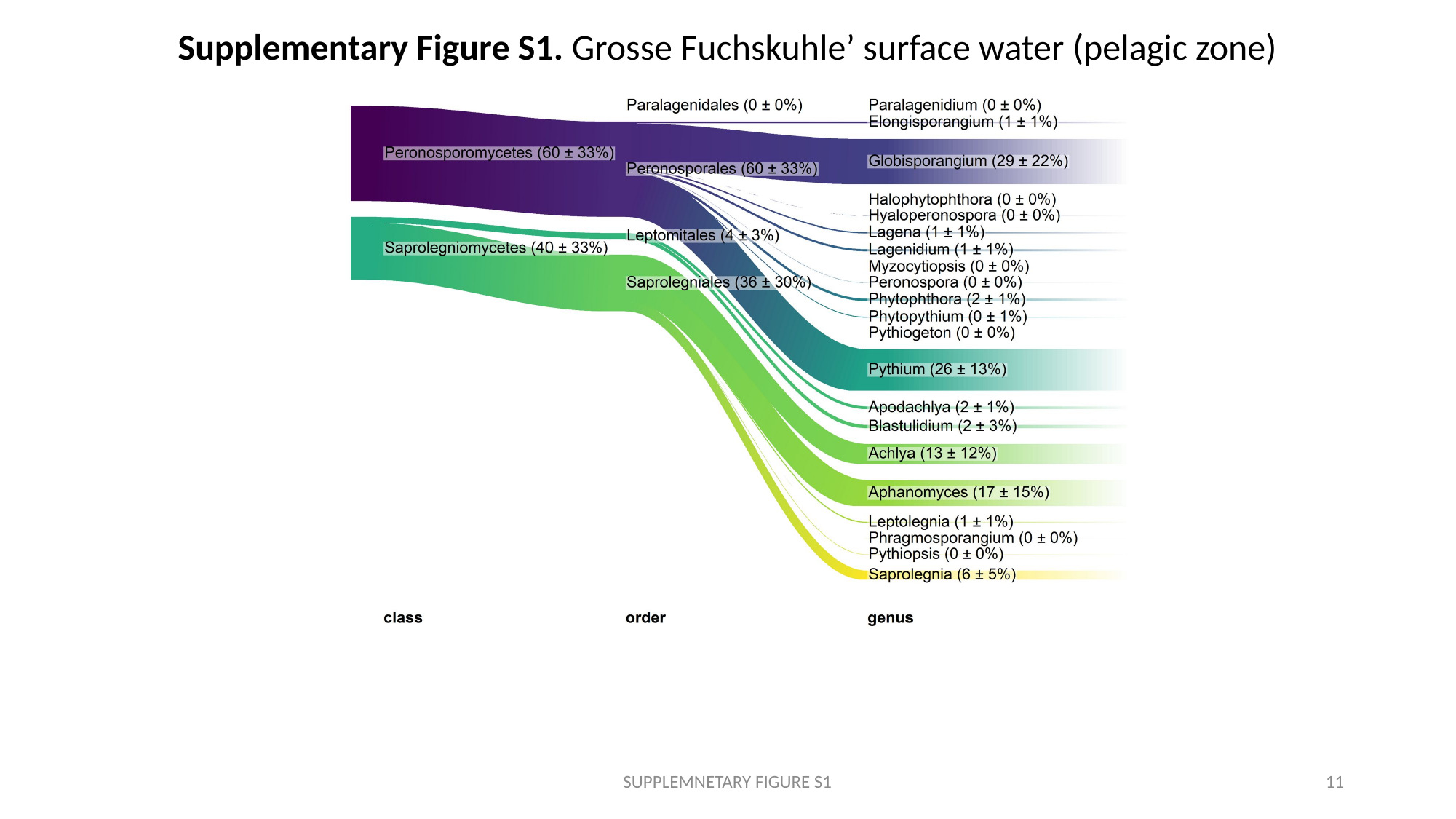

Supplementary Figure S1. Grosse Fuchskuhle’ surface water (pelagic zone)
SUPPLEMNETARY FIGURE S1
11

## Slide 12
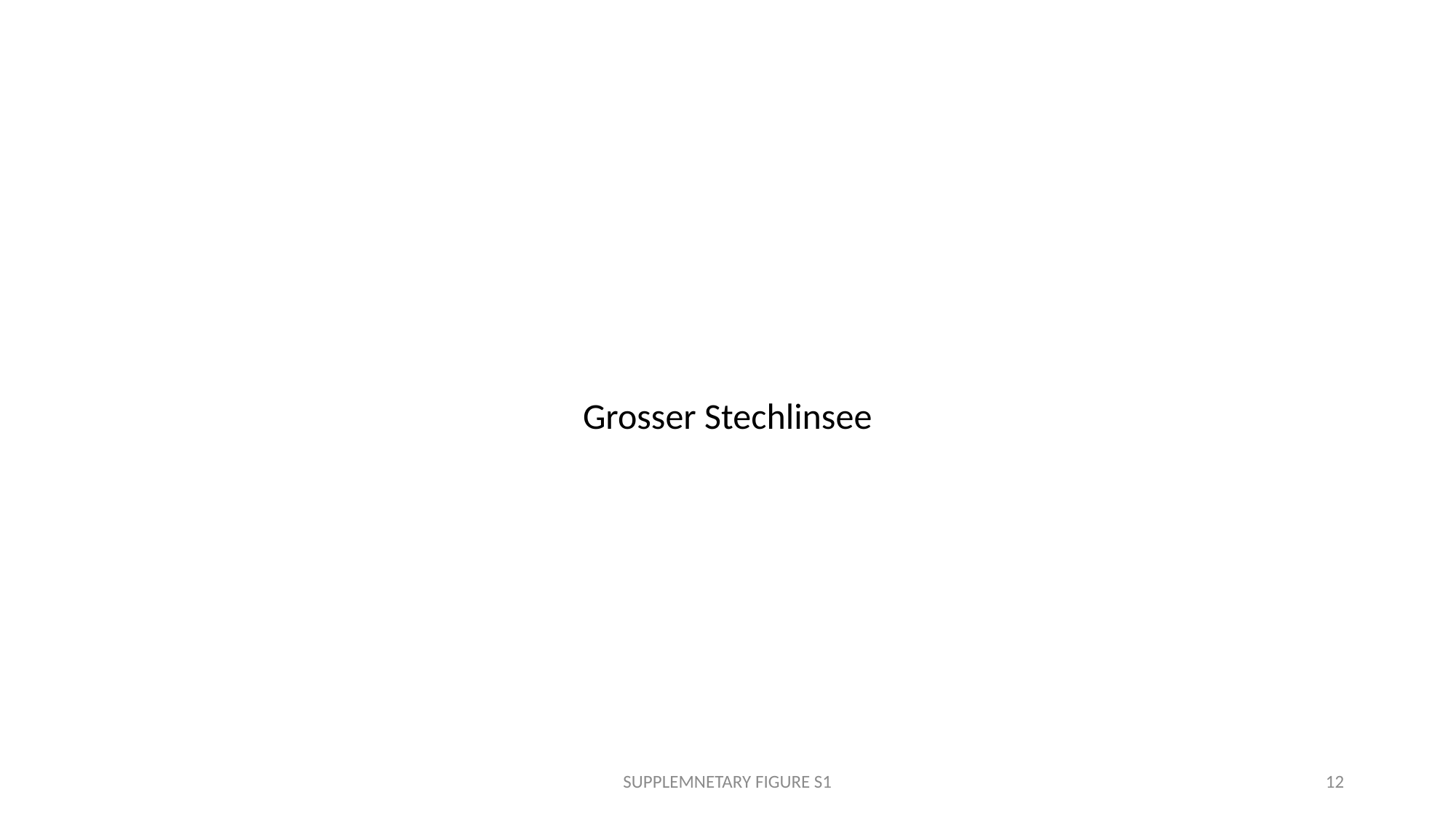

Grosser Stechlinsee
SUPPLEMNETARY FIGURE S1
12

## Slide 13
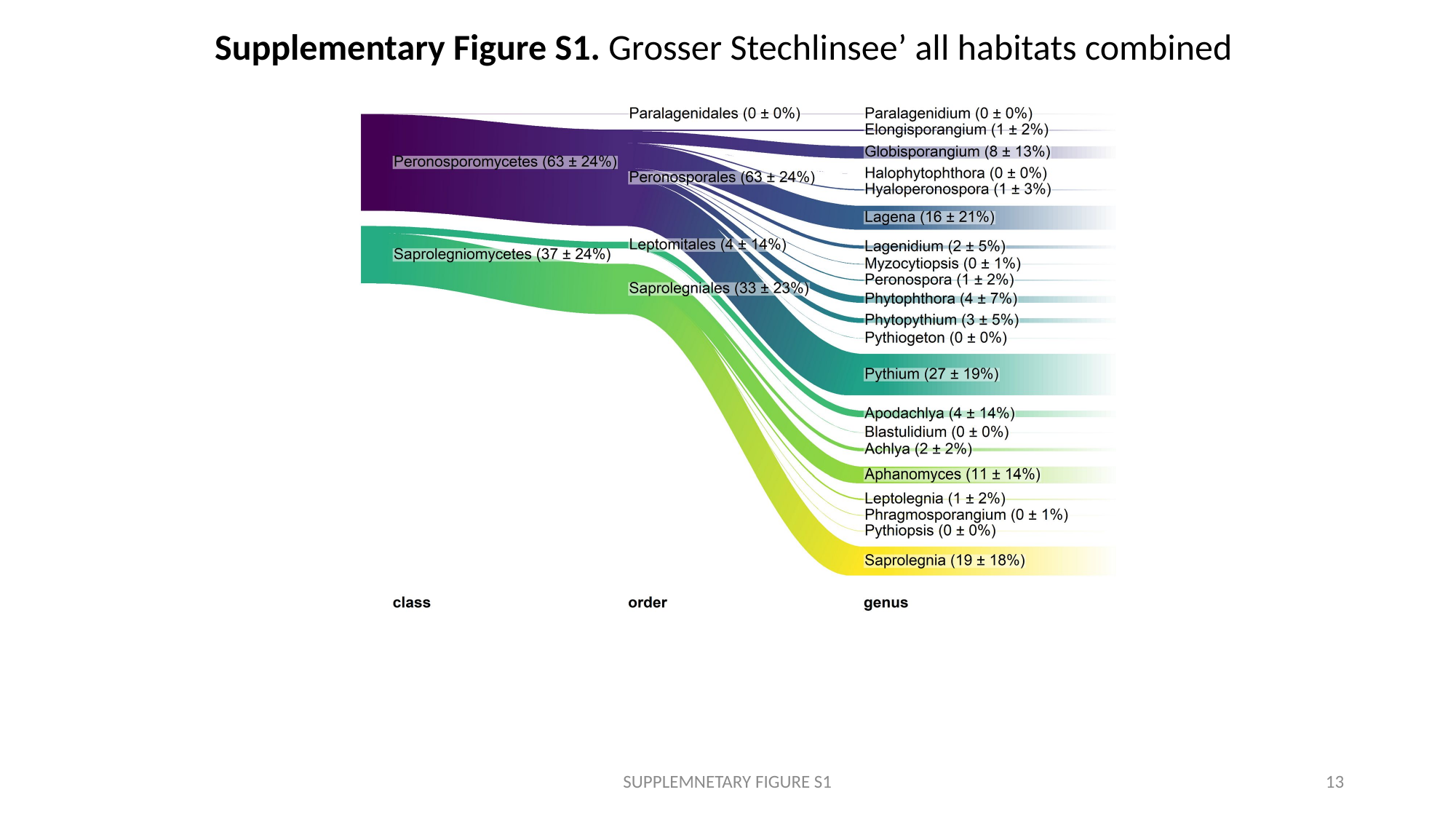

Supplementary Figure S1. Grosser Stechlinsee’ all habitats combined
SUPPLEMNETARY FIGURE S1
13

## Slide 14
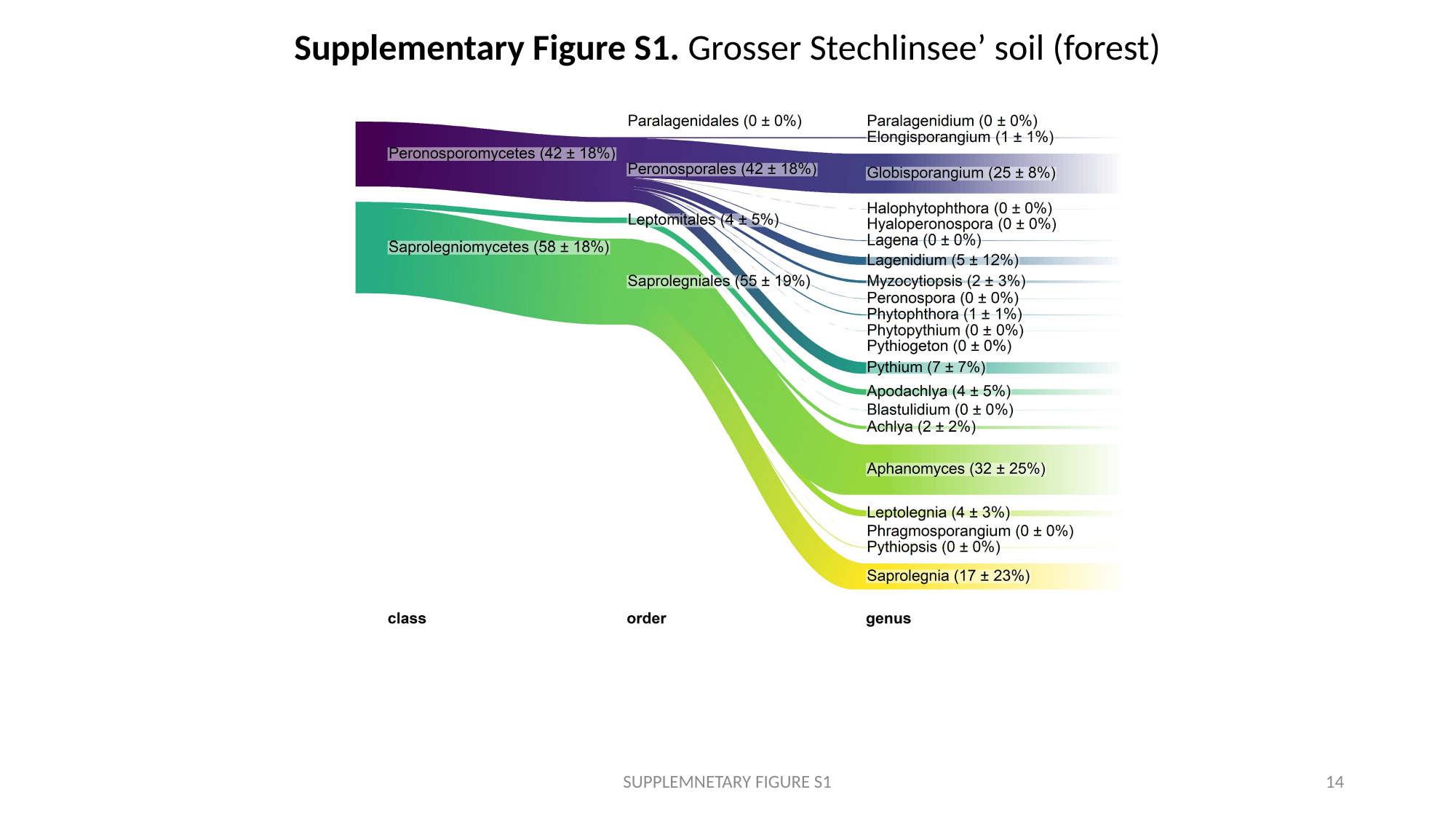

Supplementary Figure S1. Grosser Stechlinsee’ soil (forest)
SUPPLEMNETARY FIGURE S1
14

## Slide 15
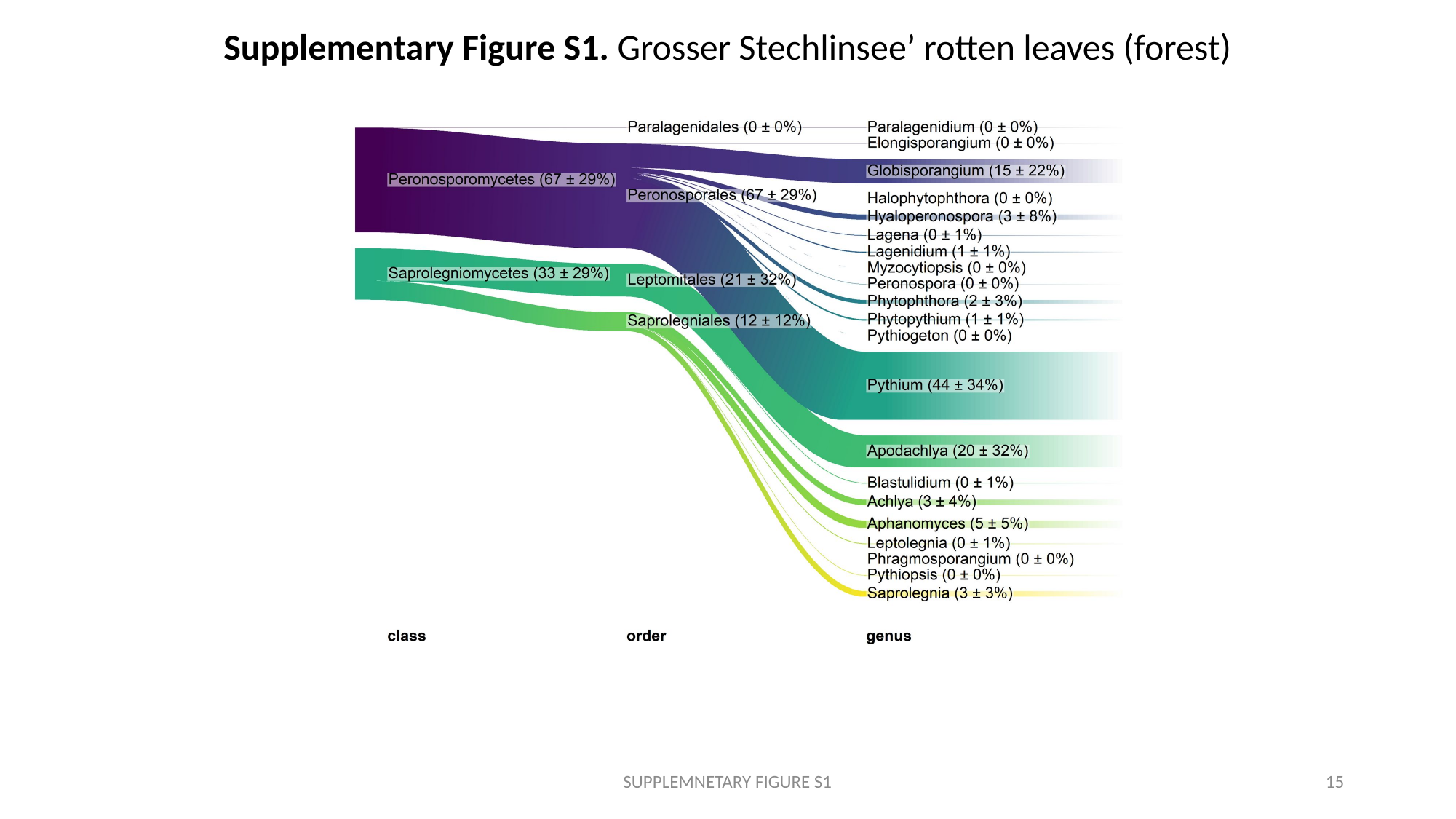

Supplementary Figure S1. Grosser Stechlinsee’ rotten leaves (forest)
SUPPLEMNETARY FIGURE S1
15

## Slide 16
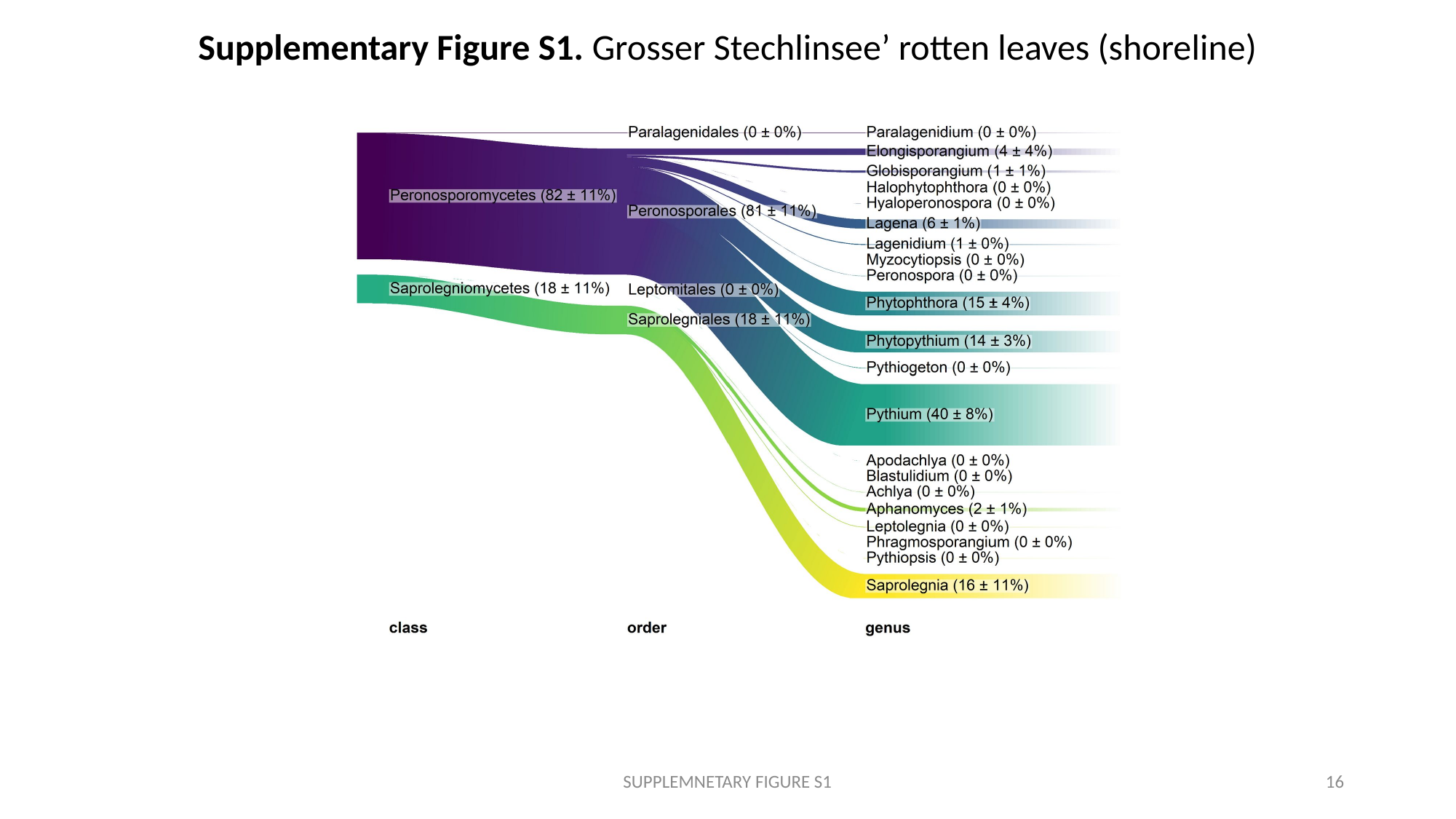

Supplementary Figure S1. Grosser Stechlinsee’ rotten leaves (shoreline)
SUPPLEMNETARY FIGURE S1
16

## Slide 17
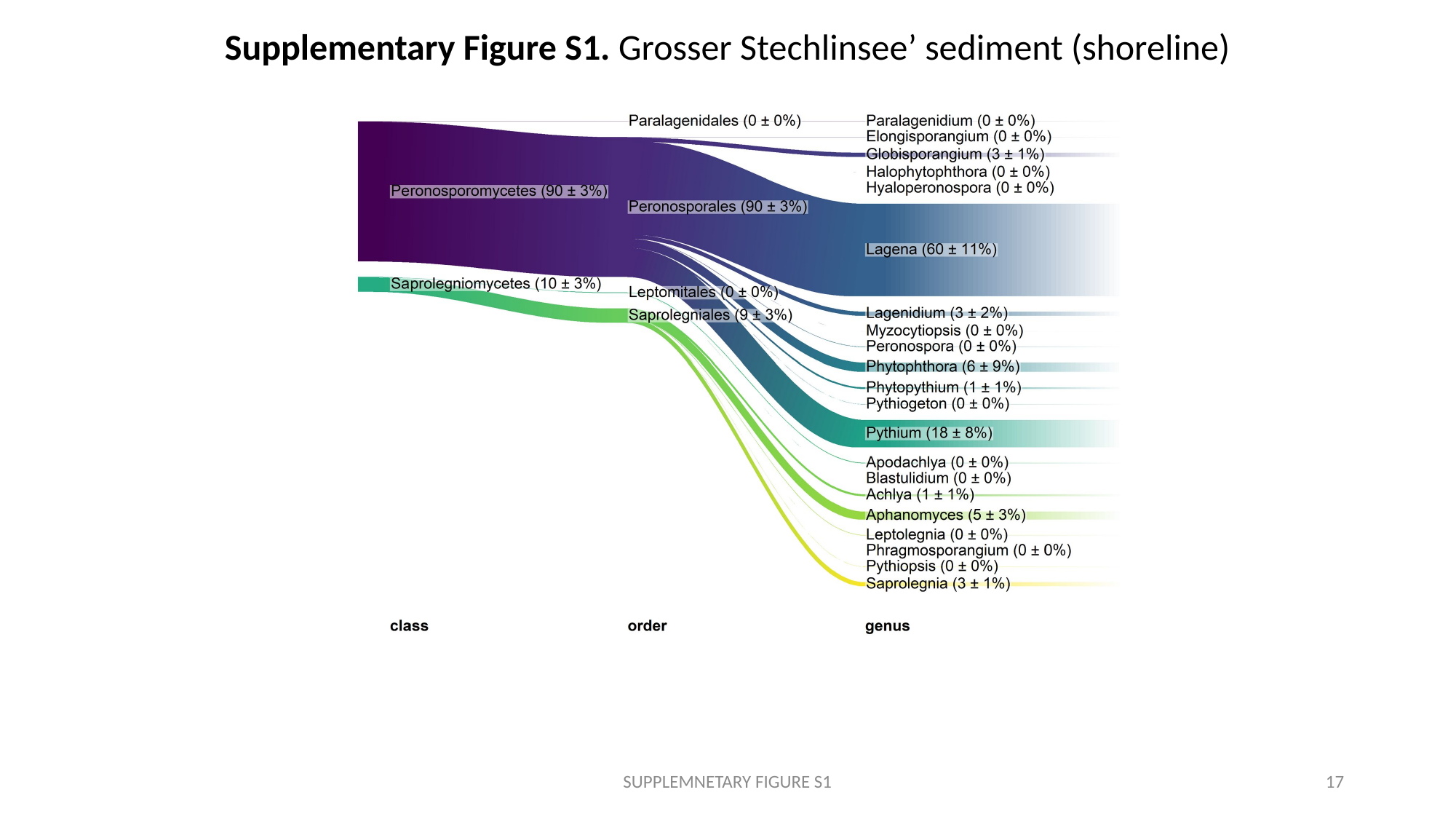

Supplementary Figure S1. Grosser Stechlinsee’ sediment (shoreline)
SUPPLEMNETARY FIGURE S1
17

## Slide 18
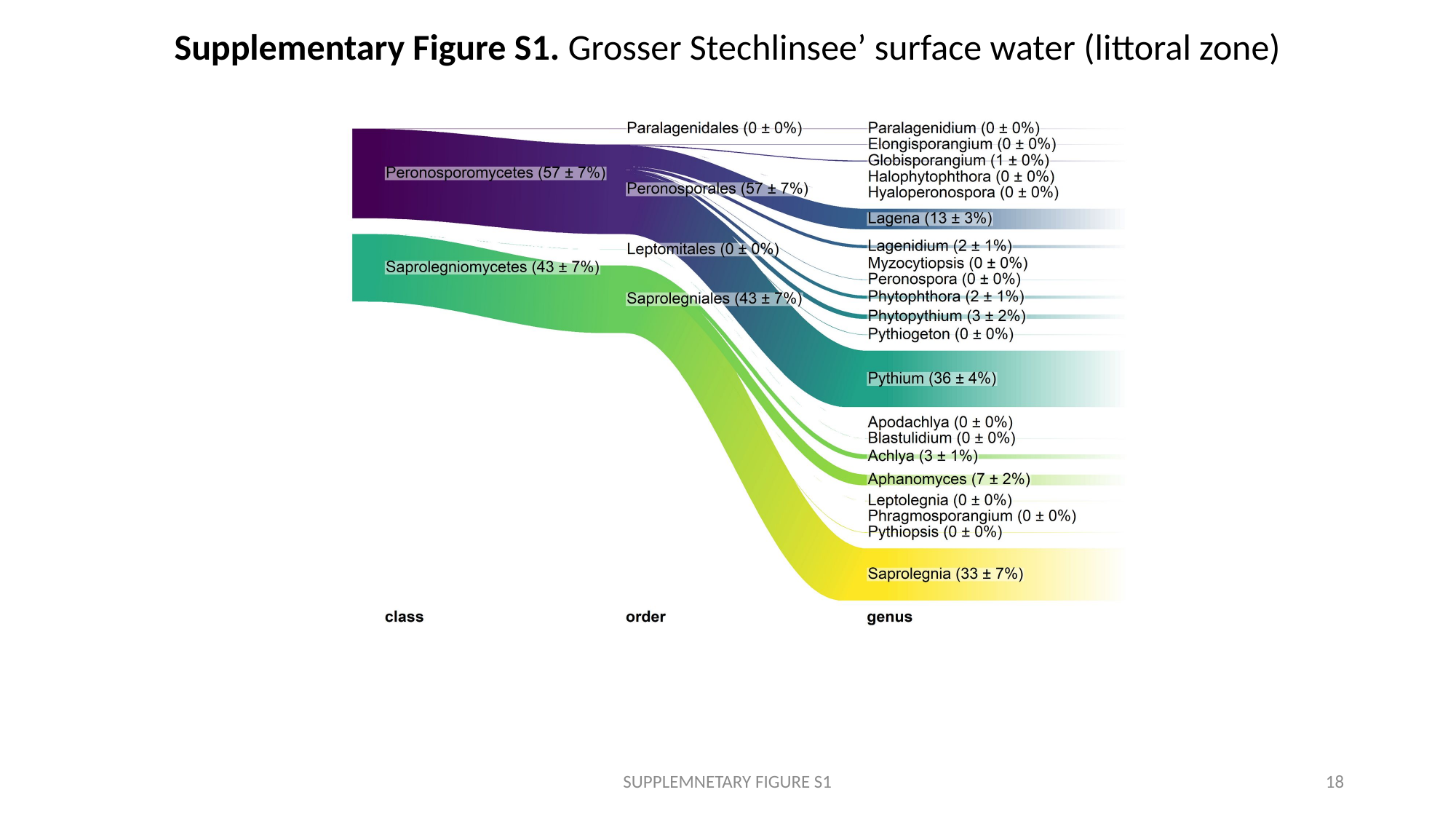

Supplementary Figure S1. Grosser Stechlinsee’ surface water (littoral zone)
SUPPLEMNETARY FIGURE S1
18

## Slide 19
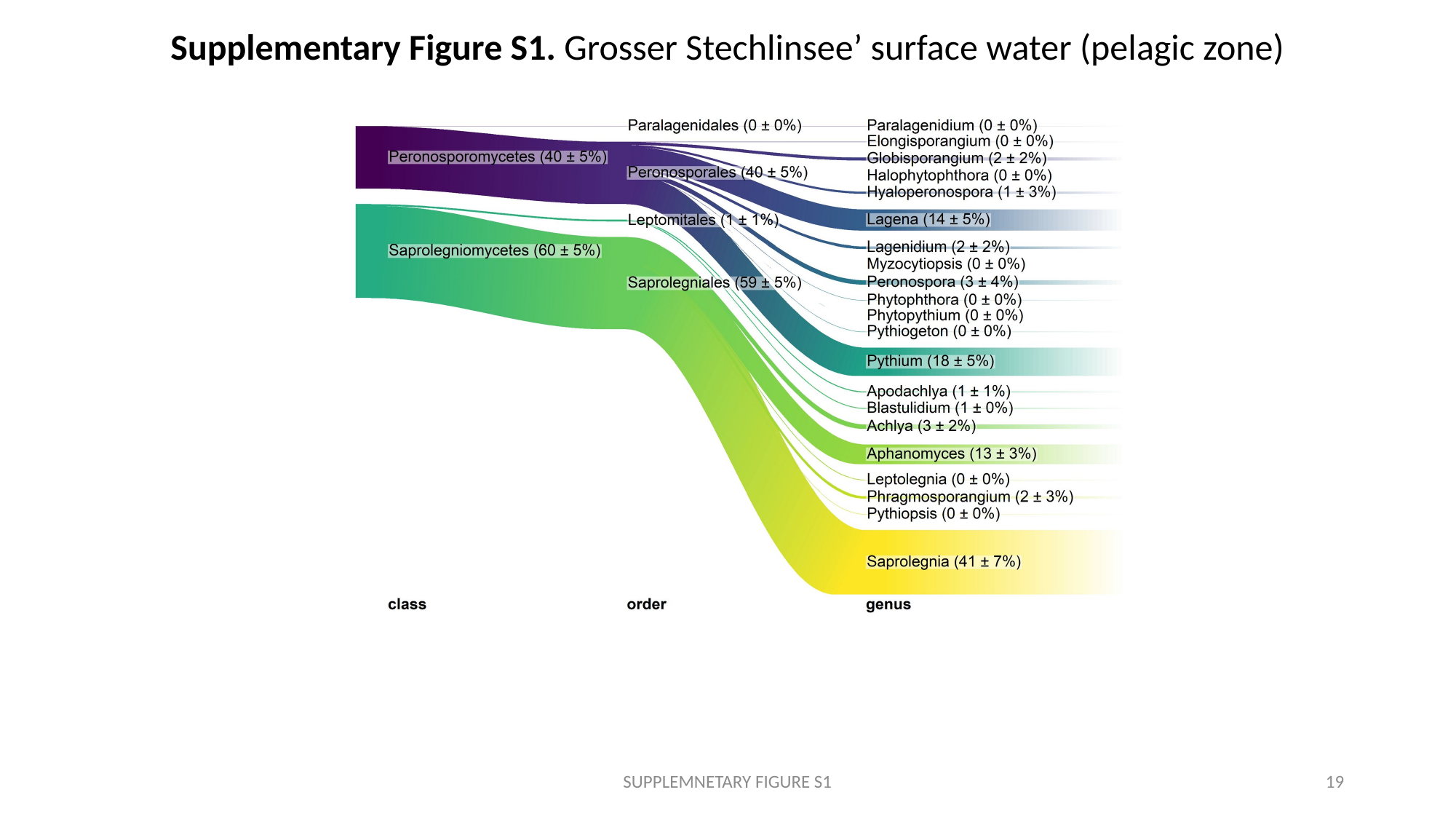

Supplementary Figure S1. Grosser Stechlinsee’ surface water (pelagic zone)
SUPPLEMNETARY FIGURE S1
19
