# Supplementary material for: A glimpse into Oomycota diversity in freshwater lakes and adjacent forests using a metabarcoding approach
Source: Sci Rep. 2025 May 31;15:19124. doi: 10.1038/s41598-025-01727-3 (PMC12126517; doi:10.1038/s41598-025-01727-3)
Supplement: Supplementary file 1 — Supplementary Material 1 [file 41598_2025_1727_MOESM1_ESM.zip › Supplementary Figure S2.pptx]

## Slide 1
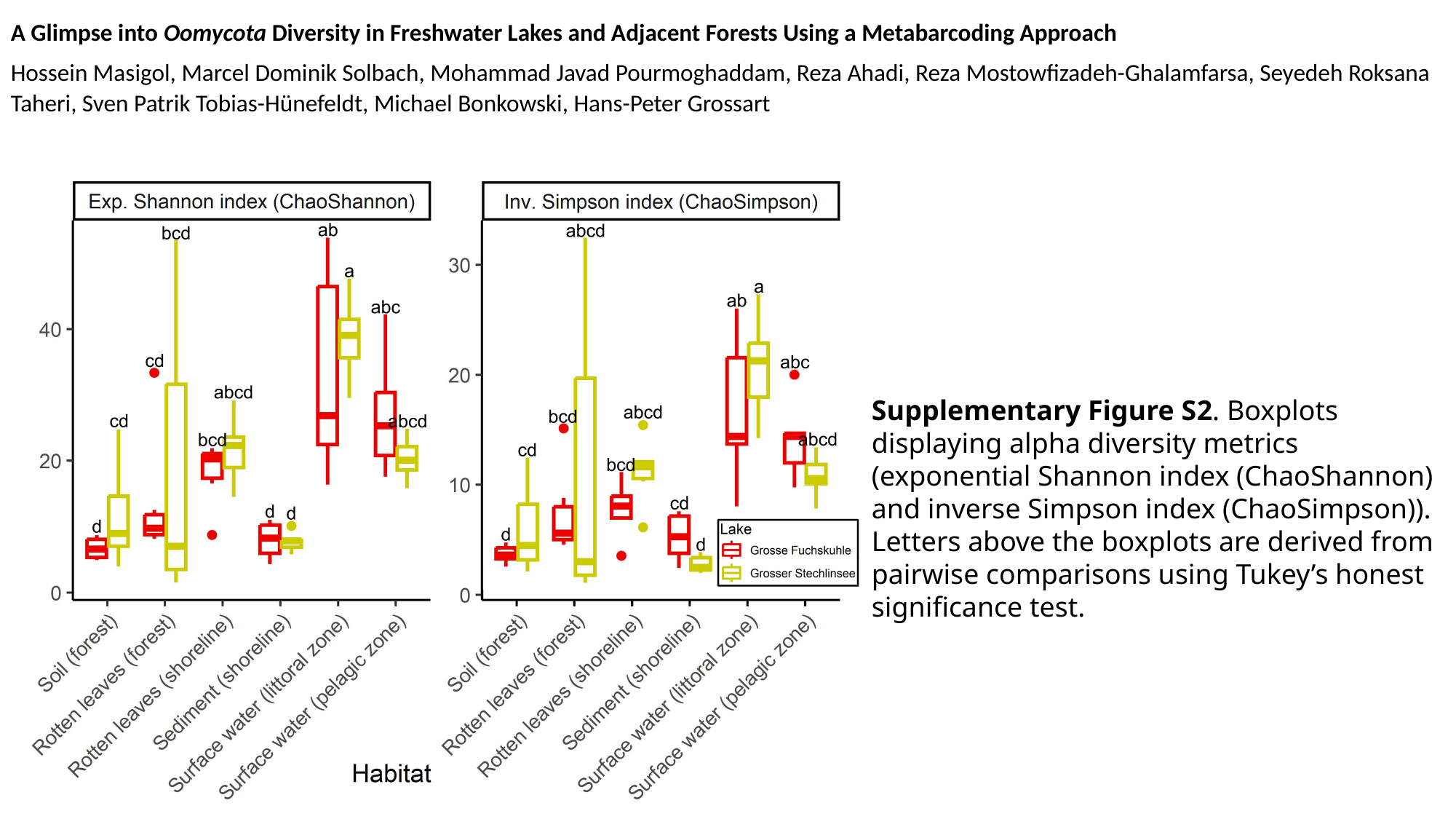

A Glimpse into Oomycota Diversity in Freshwater Lakes and Adjacent Forests Using a Metabarcoding Approach
Hossein Masigol, Marcel Dominik Solbach, Mohammad Javad Pourmoghaddam, Reza Ahadi, Reza Mostowfizadeh-Ghalamfarsa, Seyedeh Roksana Taheri, Sven Patrik Tobias-Hünefeldt, Michael Bonkowski, Hans-Peter Grossart
Supplementary Figure S2. Boxplots displaying alpha diversity metrics (exponential Shannon index (ChaoShannon) and inverse Simpson index (ChaoSimpson)). Letters above the boxplots are derived from pairwise comparisons using Tukey’s honest significance test.
