# Supplementary material for: A glimpse into Oomycota diversity in freshwater lakes and adjacent forests using a metabarcoding approach
Source: Sci Rep. 2025 May 31;15:19124. doi: 10.1038/s41598-025-01727-3 (PMC12126517; doi:10.1038/s41598-025-01727-3)
Supplement: Supplementary file 1 — Supplementary Material 1 [file 41598_2025_1727_MOESM1_ESM.zip › Supplementary Figure S3.pptx]

## Slide 1
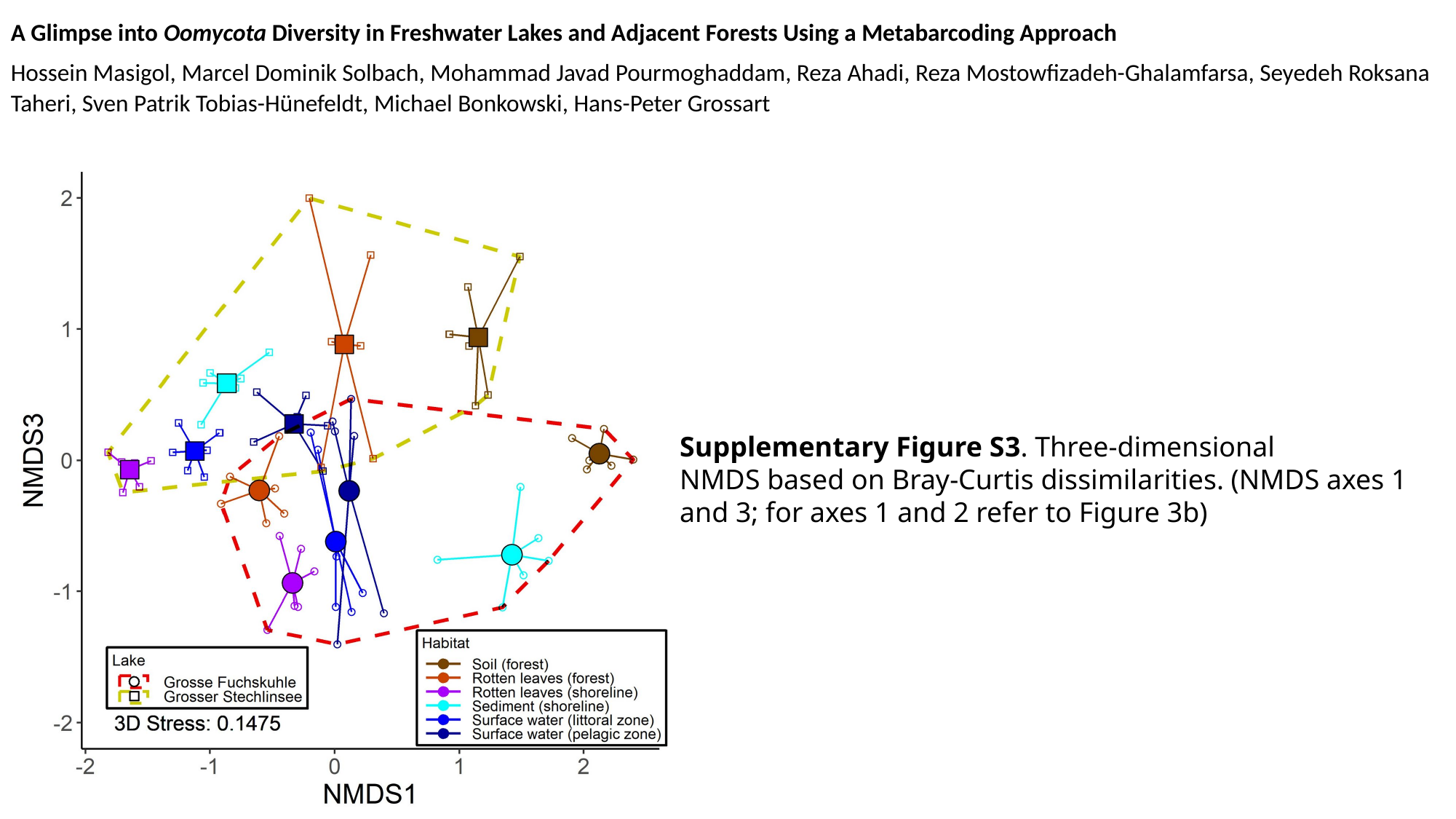

A Glimpse into Oomycota Diversity in Freshwater Lakes and Adjacent Forests Using a Metabarcoding Approach
Hossein Masigol, Marcel Dominik Solbach, Mohammad Javad Pourmoghaddam, Reza Ahadi, Reza Mostowfizadeh-Ghalamfarsa, Seyedeh Roksana Taheri, Sven Patrik Tobias-Hünefeldt, Michael Bonkowski, Hans-Peter Grossart
Supplementary Figure S3. Three-dimensional
NMDS based on Bray-Curtis dissimilarities. (NMDS axes 1 and 3; for axes 1 and 2 refer to Figure 3b)
