# Supplementary material for: A glimpse into Oomycota diversity in freshwater lakes and adjacent forests using a metabarcoding approach
Source: Sci Rep. 2025 May 31;15:19124. doi: 10.1038/s41598-025-01727-3 (PMC12126517; doi:10.1038/s41598-025-01727-3)
Supplement: Supplementary file 1 — Supplementary Material 1 [file 41598_2025_1727_MOESM1_ESM.zip › Supplementary Figure S4.pptx]

## Slide 1
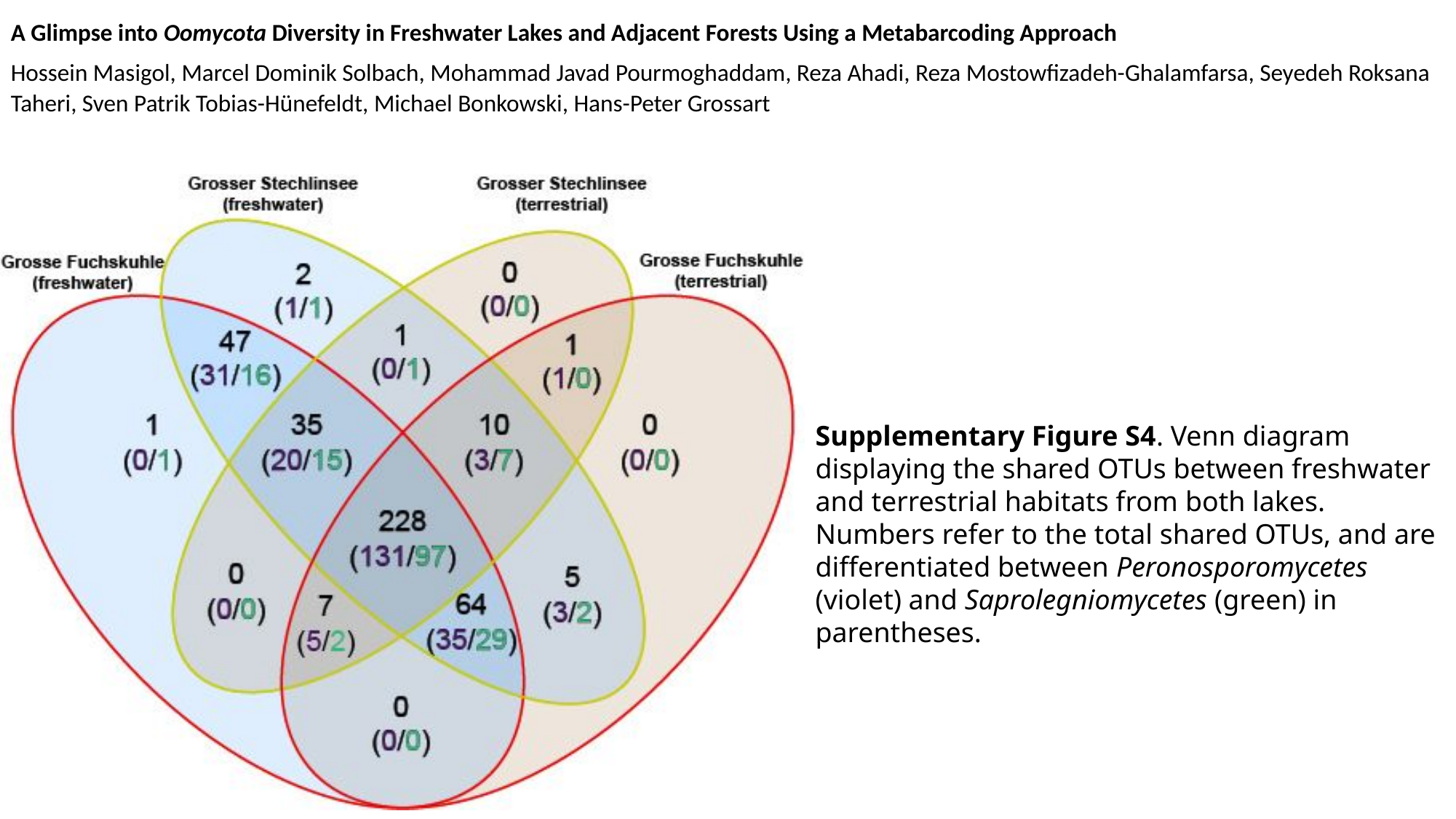

A Glimpse into Oomycota Diversity in Freshwater Lakes and Adjacent Forests Using a Metabarcoding Approach
Hossein Masigol, Marcel Dominik Solbach, Mohammad Javad Pourmoghaddam, Reza Ahadi, Reza Mostowfizadeh-Ghalamfarsa, Seyedeh Roksana Taheri, Sven Patrik Tobias-Hünefeldt, Michael Bonkowski, Hans-Peter Grossart
Supplementary Figure S4. Venn diagram displaying the shared OTUs between freshwater and terrestrial habitats from both lakes. Numbers refer to the total shared OTUs, and are differentiated between Peronosporomycetes (violet) and Saprolegniomycetes (green) in parentheses.
