# Supplementary material for: A glimpse into Oomycota diversity in freshwater lakes and adjacent forests using a metabarcoding approach
Source: Sci Rep. 2025 May 31;15:19124. doi: 10.1038/s41598-025-01727-3 (PMC12126517; doi:10.1038/s41598-025-01727-3)
Supplement: Supplementary file 1 — Supplementary Material 1 [file 41598_2025_1727_MOESM1_ESM.zip › Supplementary Table S1.docx]

| **A Glimpse into Oomycota Diversity in Freshwater Lakes and Adjacent Forests Using a Metabarcoding Approach**  Hossein Masigol, Marcel Dominik Solbach, Mohammad Javad Pourmoghaddam, Reza Ahadi, Reza Mostowfizadeh-Ghalamfarsa, Seyedeh Roksana Taheri, Sven Patrik Tobias-Hünefeldt, Michael Bonkowski, Hans-Peter Grossart | | | | | |
| --- | --- | --- | --- | --- | --- |
| **Supplementary Table S1.** Isolation and accession numbers of sequences used in the phylogenetic analyses. Isolates/sequences in bold were isolated/sequenced in present study. N/A: not available. | | | | | |
| **References** | **GenBank Accessions (ITS)** | **Substrate** | **Country** | **Strain Number** | **Species** |
| Robideau et al.^1^ | HQ643082 | N/A | France | CBS 383.79 | *Achlya ambisexualis* |
| Robideau et al.^1^ | HQ643083 | N/A | USA | CBS 101.50 | *Achlya ambisexualis* |
| Robideau et al.^1^ | HQ643088 | N/A | Sweden | CBS 100.42 | *Achlya bisexualis* |
| Robideau et al.^1^ | HQ643087 | N/A | USA | CBS 102.62 | *Achlya bisexualis* |
| Robideau et al.^1^ | HQ643089 | N/A | Nigeria | CBS 544.67 | *Achlya caroliniana* |
| Steciow et al.^2^ | KP098355 | N/A | Argentina | VKMF1904 | *Achlya debaryana* |
| Steciow et al.^2^ | KP098352 | N/A | Argentina | VKMF1904 | *Achlya debaryana* |
| Robideau et al.^1^ | HQ643093 | N/A | Nigeria | CBS 546.67 | *Achlya dubia* |
| Robideau et al.^1^ | HQ643096 | N/A | N/A | CBS 107.35 | *Achlya flagellata* |
| Robideau et al.^1^ | HQ643099 | N/A | USA | CBS 420.65 | *Achlya heterosexualis* |
| Robideau et al.^1^ | HQ643100 | N/A | USA | CBS 419.65 | *Achlya heterosexualis* |
| Not published | OK270986 | N/A | Brazil | CCIBt4421 | *Achlya klebsiana* |
| **This study** | **PP995782** | **Sediment (shoreline)** | **Germany** | **P2B01** | ***Achlya klebsiana*** |
| **This study** | **PP995783** | **Sediment (shoreline)** | **Germany** | **P2D04** | ***Achlya klebsiana*** |
| Not published | OK270990 | N/A | Brazil | CCIBt4419 | *Achlya proliferoides* |
| **This study** | **PP995820** | **Surface water (pelagic zone)** | **Germany** | **P1H08** | ***Achlya* sp.** |
| **This study** | **PP995821** | **Surface water (littoral zone)** | **Germany** | **P2A02** | ***Achlya* sp.** |
| **This study** | **PP995822** | **Surface water (pelagic zone)** | **Germany** | **P2E06** | ***Achlya* sp.** |
| **This study** | **PP995823** | **Sediment (shoreline)** | **Germany** | **P2F04** | ***Achlya* sp.** |
| Choi et al.^3^ | DQ418496 | *Diplotaxis erucoides* | Palestine | BPI184862 | *Albugo candida* |
| Choi et al.^3^ | DQ418493 | *Heliophila meyeri* | South Africa | BPI184888 | *Albugo candida* |
| LéVesque and de Cock^4^ | AY598650 | *Rheum rhaponticum* | USA | CBS 285.31 | *Elongisporangium anandrum* |
| LéVesque and de Cock^4^ | AY598651 | *Pinus taeda* (roots) | USA | CBS 406.72 | *Elongisporangium dimorphum* |
| LéVesque and de Cock^4^ | AY598653 | *Rumex acetosella* | USA | CBS 393.54 | *Elongisporangium helicandrum* |
| LéVesque and de Cock^4^ | AY598708 | soil under *Pinus* sp. | USA | CBS 157.69 | *Elongisporangium undulatum* |
| **This study** | **PP995784** | **Surface water (littoral zone)** | **Germany** | **P1F09** | ***Elongisporangium undulatum*** |
| **This study** | **PP995785** | **Surface water (pelagic zone)** | **Germany** | **P1F10** | ***Elongisporangium undulatum*** |
| **This study** | **PP995786** | **Rotten leaves (shoreline)** | **Germany** | **P1H10** | ***Elongisporangium undulatum*** |
| **This study** | **PP995787** | **Surface water (pelagic zone)** | **Germany** | **P2C11** | ***Elongisporangium undulatum*** |
| **This study** | **PP995788** | **Surface water (pelagic zone)** | **Germany** | **P2E05** | ***Elongisporangium undulatum*** |
| Robideau et al.^1^ | HQ643473 | N/A | USA | Lev3004 | *Globisporangium attrantheridium* |
| Robideau et al.^1^ | HQ643572 | N/A | Netherlands | CBS 266.38 | *Globisporangium intermedium* |
| Robideau et al.^1^ | HQ643684 | N/A | Netherlands | CBS 574.80 | *Globisporangium macrosporum* |
| **Table S1.** Continued | | | | | |
| **References** | **GenBank Accessions (ITS)** | **Substrate** | **Country** | **Strain Number** | **Species** |
| **This study** | **PP995824** | **Rotten leaves (forest)** | **Germany** | **P1B05** | ***Globisporangium attrantheridium*** |
| **This study** | **PP995825** | **Soil (forest)** | **Germany** | **P2F01** | ***Globisporangium attrantheridium*** |
| Steciow et al.^5^ | KF766944 | N/A | Argentina | CBS 359.35 | *Leptolegnia caudata* |
| Steciow et al.^5^ | KF766943 | N/A | Argentina | CBS 680.69 | *Leptolegnia caudata* |
| Steciow et al.^5^ | KF766942 | N/A | Argentina | CBS 113431 | *Leptolegnia caudata* |
| **This study** | **PP995789** | **Sediment (shoreline)** | **Germany** | **P2H02** | ***Leptolegnia caudata*** |
| Robideau et al.^1^ | HQ643373 | N/A | France | CBS 112544 | *Phytopythium carbonicum* |
| Robideau et al.^1^ | HQ643374 | N/A | N/A | CBS 259.30 | *Phytopythium chamaehyphon* |
| Broders et al.^6^ | EU339312 | *Glycine max* | USA | OH382 | *Phytopythium delawarense* |
| Robideau et al.^1^ | HQ643382 | N/A | USA | CBS 167.68 | *Phytopythium helicoides* |
| Robideau et al.^1^ | HQ643391 | N/A | Germany | ADC9762 | *Phytopythium montanum* |
| Jankowiak et al.^7^ | KC602493 | Soil (Quercus robur) | Poland | 451HR08 | *Phytopythium* sp. |
| Jankowiak et al.^7^ | KC602492 | Soil (Quercus robur) | Poland | 68HR09 | *Phytopythium* sp. |
| **This study** | **PP995790** | **Rotten leaves (shoreline)** | **Germany** | **P2D05** | ***Phytopythium* sp.** |
| **This study** | **PP995791** | **Rotten leaves (forest)** | **Germany** | **P1H11** | ***Phytopythium* sp.** |
| **This study** | **PP995826** | **Rotten leaves (shoreline)** | **Germany** | **P2A04** | ***Phytopythium* sp.** |
| Garibaldi et al.^8^ | JX462954 | *Beta vulgaris* | Italy | Py7/10 | *Pythium aphanidermatum* |
| Robideau et al.^1^ | HQ643445 | N/A | USA | CBS 215.80 | *Pythium aquatile* |
| Robideau et al.^1^ | HQ643447 | N/A | Canada | CBS 263.38 | *Pythium aristosporum* |
| Robideau et al.^1^ | HQ643494 | N/A | USA | CBS 842.68 | *Pythium catenulatum* |
| Robideau et al.^1^ | HQ643522 | N/A | N/A | CBS 314.33 | *Pythium deliense* |
| Robideau et al.^1^ | HQ643509 | N/A | USA | CBS 223.88 | *Pythium conidiophorum* |
| Robideau et al.^1^ | HQ643566 | N/A | USA | CBS 168.68 | *Pythium inflatum* |
| Robideau et al.^1^ | HQ643526 | N/A | Australia | CBS 155.64 | *Pythium dissimile* |
| Robideau et al.^1^ | HQ643545 | N/A | Jamaica | CBS 327.62 | *Pythium graminicola* |
| LéVesque and de Cock^4^ | AY598688 | Soil | UK | CBS 222.88 | *Pythium lutarium* |
| LéVesque and de Cock^4^ | AY598689 | Soil | UK | CBS 750.96 | *Pythium marinum* |
| Bala et al.^9^ | FJ655174 | *Cucumis sativus* | Canada | DAOM BR632 | *Pythium oopapillum* |
| LéVesque and de Cock^4^ | AY598687 | Soil | UK | CBS 227.88 | *Pythium pachycaule* |
| **This study** | **PP995792** | **Sediment (shoreline)** | **Germany** | **P1E03** | ***Pythium pachycaule*** |
| **This study** | **PP995793** | **Sediment (shoreline)** | **Germany** | **P1H05** | ***Pythium pachycaule*** |
| **This study** | **PP995794** | **Surface water (littoral zone)** | **Germany** | **P2A06** | ***Pythium pachycaule*** |
| **This study** | **PP995795** | **Sediment (shoreline)** | **Germany** | **P2A08** | ***Pythium pachycaule*** |
| Rahman et al.^10^ | AB998878 | Soil | Japan | CBS 139278 | *Pythium rishiriense* |
| **This study** | **PP995796** | **Surface water (pelagic zone)** | **Germany** | **P1E02** | ***Pythium rishiriense*** |
| **This study** | **PP995797** | **Surface water (littoral zone)** | **Germany** | **P1F11** | ***Pythium rishiriense*** |
| **This study** | **PP995798** | **Surface water (pelagic zone)** | **Germany** | **P1H01** | ***Pythium rishiriense*** |
| **Table S1.** Continued | | | | | |
| **References** | **GenBank Accessions (ITS)** | **Substrate** | **Country** | **Strain Number** | **Species** |
| **This study** | **PP995799** | **Rotten leaves (shoreline)** | **Germany** | **P1H06** | ***Pythium rishiriense*** |
| **This study** | **PP995800** | **Surface water (pelagic zone)** | **Germany** | **P1H09** | ***Pythium rishiriense*** |
| **This study** | **PP995801** | **Surface water (pelagic zone)** | **Germany** | **P2A03** | ***Pythium rishiriense*** |
| **This study** | **PP995802** | **Surface water (littoral zone)** | **Germany** | **P2A07** | ***Pythium rishiriense*** |
| **This study** | **PP995803** | **Surface water (littoral zone)** | **Germany** | **P2A09** | ***Pythium rishiriense*** |
| **This study** | **PP995804** | **Rotten leaves (shoreline)** | **Germany** | **P2A10** | ***Pythium rishiriense*** |
| **This study** | **PP995805** | **Rotten leaves (forest)** | **Germany** | **P2A12** | ***Pythium rishiriense*** |
| **This study** | **PP995806** | **Surface water (pelagic zone)** | **Germany** | **P2B03** | ***Pythium rishiriense*** |
| **This study** | **PP995807** | **Surface water (littoral zone)** | **Germany** | **P2B05** | ***Pythium rishiriense*** |
| **This study** | **PP995808** | **Surface water (littoral zone)** | **Germany** | **P2B06** | ***Pythium rishiriense*** |
| **This study** | **PP995809** | **Rotten leaves (shoreline)** | **Germany** | **P2B08** | ***Pythium rishiriense*** |
| **This study** | **PP995810** | **Surface water (pelagic zone)** | **Germany** | **P2C10** | ***Pythium rishiriense*** |
| **This study** | **PP995811** | **Surface water (pelagic zone)** | **Germany** | **P2D02** | ***Pythium rishiriense*** |
| **This study** | **PP995812** | **Surface water (pelagic zone)** | **Germany** | **P2D06** | ***Pythium rishiriense*** |
| **This study** | **PP995813** | **Surface water (pelagic zone)** | **Germany** | **P2D08** | ***Pythium rishiriense*** |
| **This study** | **PP995814** | **Surface water (littoral zone)** | **Germany** | **P2E01** | ***Pythium rishiriense*** |
| **This study** | **PP995815** | **Surface water (pelagic zone)** | **Germany** | **P2E08** | ***Pythium rishiriense*** |
| Robideau et al.^1^ | HQ643859 | N/A | Netherlands | CBS 316.33 | *Pythium torulosum* |
| Robideau et al.^1^ | HQ643971 | N/A | Japan | CBS 699.83 | *Pythium volutum* |
| **This study** | **PP995827** | **Surface water (pelagic zone)** | **Germany** | **P1H07** | ***Pythium* sp.** |
| **This study** | **PP995828** | **Surface water (pelagic zone)** | **Germany** | **P2A11** | ***Pythium* sp.** |
| **This study** | **PP995829** | **Surface water (littoral zone)** | **Germany** | **P2B10** | ***Pythium* sp.** |
| **This study** | **PP995830** | **Surface water (littoral zone)** | **Germany** | **P2C01** | ***Pythium* sp.** |
| **This study** | **PP995831** | **Surface water (littoral zone)** | **Germany** | **P2B02** | ***Pythium* sp.** |
| **This study** | **PP995832** | **Rotten leaves (shoreline)** | **Germany** | **P2D07** | ***Pythium* sp.** |
| **This study** | **PP995833** | **Sediment (shoreline)** | **Germany** | **P2G10** | ***Pythium* sp.** |
| Sandoval-Sierra and Diéguez-Uribeondo^11^ | KR872872 | Egg | Chile | RJBCC0028 | *Saprolegnia aenigmatica* |
| Sandoval-Sierra et al.^12^ | KF718010 | River | Spain | SAP1105 | *Saprolegnia australis* |
| Sandoval-Sierra et al.^12^ | KF718043 | River | Argentina | SAP1524 | *Saprolegnia delica* |
| Sandoval-Sierra et al.^12^ | KF717815 | River | Spain | SAP1281 | *Saprolegnia diclina* |
| Sandoval-Sierra et al.^12^ | KF717954 | River | Ecuador | SAP1234 | *Saprolegnia ferax* |
| **This study** | **PP995834** | **Surface water (pelagic zone)** | **Germany** | **P1B01** | ***Saprolegnia* *ferax*** |
| **This study** | **PP995835** | **Surface water (pelagic zone)** | **Germany** | **P1G07** | ***Saprolegnia ferax*** |
| **This study** | **PP995836** | **Surface water (pelagic zone)** | **Germany** | **P2E03** | ***Saprolegnia ferax*** |
| Sandoval-Sierra et al.^12^ | KF718048 | River | UK | SAP1486 | *Saprolegnia litoralis* |
| Sandoval-Sierra et al.^12^ | KF717876 | River | Ecuador | SAP1381 | *Saprolegnia parasitica* |
| **Table S1.** Continued | | | | | |
| **References** | **GenBank Accessions (ITS)** | **Substrate** | **Country** | **Strain Number** | **Species** |
| Sandoval-Sierra and Diéguez-Uribeondo^11^ | KR872846 | Egg | Spain | RJBCC0002 | *Saprolegnia racemosa* |
| Sandoval-Sierra et al.^12^ | KF718124 | Drainage muck | USA | SAP1293 | *Saprolegnia subterranea* |
| **This study** | **PP995816** | **Sediment (shoreline)** | **Germany** | **P1E04** | ***Saprolegnia* sp.** |
| **This study** | **PP995817** | **Rotten leaves (shoreline)** | **Germany** | **P1F12** | ***Saprolegnia* sp.** |
| **This study** | **PP995818** | **Rotten leaves (shoreline)** | **Germany** | **P1G08** | ***Saprolegnia* sp.** |
| **This study** | **PP995819** | **Sediment (shoreline)** | **Germany** | **P1H04** | ***Saprolegnia* sp.** |
| **This study** | **PP995837** | **Sediment (shoreline)** | **Germany** | **P1A02** | ***Saprolegnia* sp.** |
| **This study** | **PP995838** | **Surface water (littoral zone)** | **Germany** | **P2D03** | ***Saprolegnia* sp.** |
| **This study** | **PP995839** | **Sediment (shoreline)** | **Germany** | **P2E02** | ***Saprolegnia* sp.** |
| **This study** | **PP995840** | **Rotten leaves (shoreline)** | **Germany** | **P2F02** | ***Saprolegnia* sp.** |
| **This study** | **PP995841** | **Rotten leaves (shoreline)** | **Germany** | **P2E04** | ***Saprolegnia* sp.** |
| **This study** | **PP995842** | **Surface water (littoral zone)** | **Germany** | **P1A01** | ***Saprolegnia* sp.** |
| **This study** | **PP995843** | **Sediment (shoreline)** | **Germany** | **P1A09** | ***Saprolegnia* sp.** |
| **This study** | **PP995844** | **Surface water (pelagic zone)** | **Germany** | **P2D10** | ***Saprolegnia* sp.** |
| **This study** | **PP995845** | **Surface water (littoral zone)** | **Germany** | **P2D11** | ***Saprolegnia* sp.** |

**References**

1. Robideau, G. P. *et al*. DNA barcoding of oomycetes with cytochrome c oxidase subunit I and internal transcribed spacer. *Molecular ecology resources*, **11(6)**, 1002–1011 (2011).

2. Steciow, M. M., Lara, E., Paul, C., Pillonel, A., & Belbahri, L. Multiple barcode assessment within the *Saprolegnia-Achlya* clade (Saprolegniales, Oomycota, Straminipila) brings order in a neglected group of pathogens. *IMA fungus*, **5**, 439–448 (2014).

3. Choi, Y. J., Hong, S. B., & Shin, H. D. Genetic diversity within the *Albugo candida* complex (Peronosporales, Oomycota) inferred from phylogenetic analysis of ITS rDNA and COX2 mtDNA sequences. *Molecular phylogenetics and evolution*, **40(2)**, 400–409 (2006).

4. Levesque, C. A., & De Cock, A. W. Molecular phylogeny and taxonomy of the genus *Pythium*. *Mycological research*, **108(12)**, 1363–1383 (2004).

5. Steciow, M. M. *et al*. Incipient loss of flagella in the genus *Geolegnia*: the emergence of a new clade within Leptolegnia?. *IMA fungus*, **4**, 169–175 (2013).

6. Broders, K. D., Lipps, P. E., Ellis, M. L., & Dorrance, A. E. *Pythium delawarii*—A new species isolated from soybean in Ohio. *Mycologia*, **101(2)**, 232–238 (2009).

7. Jankowiak, R., Stepniewska, H., & Bilanski, P. Notes on some *Phytopythium* and *Pythium* species occurring in oak forests in southern Poland. *Acta Mycologica*, **50(1)** (2015).

8. Garibaldi, A., Gilardi, G., Ortu, G., & Gullino, M. L. First report of damping-off caused by *Pythium aphanidermatum* on leaf beet (*Beta vulgaris* subsp. *vulgaris*) in Italy. *Plant Disease*, **97(2)**, 292–292 (2013).

9. Bala, K., Robideau, G. P., Désaulniers, N., De Cock, A. W. A. M., & Lévesque, C. A. Taxonomy, DNA barcoding and phylogeny of three new species of *Pythium* from Canada. *Persoonia-Molecular Phylogeny and Evolution of Fungi*, **25(1)**, 22–31(2010).

10. Rahman, M. Z., Abdelzaher, H. M. A., Mingzhu, L., Motohashi, K., Suga, H., & Kageyama, K. *Pythium rishiriense* sp. nov. from water and *P. alternatum* sp. nov. from soil, two new species from Japan. *FEMS Microbiology Letters*, **362(13)**, fnv086 (2015).

11. Sandoval-Sierra, J. V., & Dieguez-Uribeondo, J. A comprehensive protocol for improving the description of Saprolegniales (Oomycota): two practical examples (Saprolegnia aenigmatica sp. nov. and Saprolegnia racemosa sp. nov.). *PloS one*, **10(7)**, e0132999 (2015).

12. Sandoval-Sierra, J. V., Martín, M. P., & Dieguez-Uribeondo, J. Species identification in the genus *Saprolegnia* (Oomycetes): defining DNA-based molecular operational taxonomic units. *Fungal biology*, **118(7)**, 559–578 (2014).
